# Task-specific network interactions across key cognitive domains

**DOI:** 10.1093/cercor/bhab531

**Published:** 2022-04-07

**Authors:** Kathleen A Williams, Ole Numssen, Gesa Hartwigsen

**Affiliations:** Lise Meitner Research Group Cognition and Plasticity, Max Planck Institute for Human Cognitive and Brain Sciences, 04103 Leipzig, Germany; Lise Meitner Research Group Cognition and Plasticity, Max Planck Institute for Human Cognitive and Brain Sciences, 04103 Leipzig, Germany; Lise Meitner Research Group Cognition and Plasticity, Max Planck Institute for Human Cognitive and Brain Sciences, 04103 Leipzig, Germany

**Keywords:** attention, connectivity, default mode network, language, semantic cognition, social cognition

## Abstract

Human cognition is organized in distributed networks in the brain. Although distinct specialized networks have been identified for different cognitive functions, previous work also emphasizes the overlap of key cognitive domains in higher level association areas. The majority of previous studies focused on network overlap and dissociation during resting states whereas task-related network interactions across cognitive domains remain largely unexplored. A better understanding of network overlap and dissociation during different cognitive tasks may elucidate flexible (re-)distribution of resources during human cognition. The present study addresses this issue by providing a broad characterization of large-scale network dynamics in three key cognitive domains. Combining prototypical tasks of the larger domains of attention, language, and social cognition with whole-brain multivariate activity and connectivity approaches, we provide a spatiotemporal characterization of multiple large-scale, overlapping networks that differentially interact across cognitive domains. We show that network activity and interactions increase with increased cognitive complexity across domains. Interaction patterns reveal a common core structure across domains as well as dissociable domain-specific network activity. The observed patterns of activation and deactivation of overlapping and strongly coupled networks provide insight beyond region-specific activity within a particular cognitive domain toward a network perspective approach across diverse key cognitive functions.

## Introduction

Cognitive functions are organized in large-scale networks in the human brain that strongly interact and partially overlap for some functions. Numerous neuroimaging experiments have elucidated such network interactions, most extensively with task-based functional magnetic resonance imaging (fMRI) (Corbetta et al. 2008; Binder and Desai 2011; Cole et al. 2013; Laird et al. 2013). Among these functions, attention, language, and social cognition represent three key human-defining facets that are central for interaction and successful communication. These cognitive faculties enable humans to adapt to ever-changing environmental conditions by flexibly allocating and reallocating cognitive resources. For instance, the ability to interactively reorient attention is essential for visuospatial navigation in a complex world and crucial for survival (Corbetta et al. 2008). Although attentional processes are shared across different species, understanding and communicating thoughts and inferring the thoughts, beliefs, and communicative intentions of others is a unique human feature. Language and social cognitive functions are closely intertwined, and some researchers have argued that, from an evolutionary perspective, language might have evolved to facilitate social exchange between larger groups (Dunbar 2004; Tomasello 2005). In particular, the ability to understand words and sentences, that is, semantic processing, is key to social interactions, planning, and problem solving (Binder and Desai 2011).

At the neural level, as measured with task-based fMRI, attention, language, and social cognition are organized in several specialized networks in the human brain (Corbetta et al. 2008; Lambon Ralph and Patterson 2008; Spreng et al. 2008; Binder et al. 2009; Schurz et al. 2020). Aside from this neural specialization, recent work also demonstrates some overlap among these processes in higher association areas (Bzdok, Hartwigsen, et al. 2016). In particular, overlap between all three domains has been demonstrated in posterior areas of the default mode network (DMN) (Numssen et al. 2021), a major brain network that was initially observed to be more active during rest and associated with mind wandering and semantic memory (Mason et al. 2007; Shehzad et al. 2009; Binder and Desai 2011; Seghier and Price 2012). The DMN consists of distributed heteromodal association areas. Tasks that elicit positive activations in the DMN involve introspective processes that are generally separated from external sensory input, for example, autobiographical memory, spontaneous thought, and social and self-referential inferences (Buckner and DiNicola 2019). More intricate task-based experimental designs and analysis methods, such as meta-analyses and activation likelihood estimation, have dissociated the larger DMN into multiple networks that fractionate across cognitive domains (Laird et al. 2009; Peer et al. 2015) and, with sufficiently large resting-state data sets, within individuals (Braga and Buckner 2017; Buckner and DiNicola 2019).

Indeed, evidence for distinct fractionating subsystems is mounting for many of the canonical resting-state networks, such as the frontoparietal control network (FPCN) (Dixon et al. 2018), and prefrontal cortical networks, including the cingulo-opercular network and salience networks (Seeley et al. 2007; Menon and Uddin 2010; Han et al. 2019). Several studies have demonstrated preferential coupling of different subnetworks during periods of continuous cognitive states, such as FPCN subnetworks to DMN or the dorsal attention network (DAN) (Dixon et al. 2017), or DMN subnetworks to language or control networks (Gordon et al. 2020). Additionally, more studies exploring whole-brain intrinsic connectivity by employing methods beyond resting-state or simple correlational techniques reveal coherent networks that are explicitly task evoked, such as the ventral attention (Corbetta et al. 2008), multiple demand (Dosenbach et al. 2006; Fedorenko et al. 2013), and semantic networks (Lambon Ralph and Patterson 2008; Binder et al. 2009; Noonan et al. 2013). More recently, it has been demonstrated that task-evoked networks of the brain are primarily shaped by intrinsic connectivity and that a stable, core architecture changes in task-general and task-specific aspects (Cole et al. 2014). Collectively, current research has expanded the original, simplistic view of a cohesive large-scale, task-negative default network that is consistently anticorrelated with task-positive networks. In essence, these studies support a more complex consideration of all of the brain’s networks, including default mode subnetworks, as spatially and temporally dynamic systems that flexibly synchronize and reorganize to accomplish cognitive functions.

Such large-scale network interactions are often missed by standard univariate analysis approaches that fail to capture concurrent activation and deactivation in brain areas (Xu et al. 2013, 2016; Xu 2015). One way to overcome this limitation is the application of spatial independent component analysis (sICA), which offers the possibility of gaining insights into whole-brain activity that simultaneously synchronizes as multiple sources to accomplish cognitive functions. However, most of the previous work employing such analytical techniques either focused on network dynamics and interactions at rest or in an isolated cognitive domain of interest. Therefore, comparisons of whole-brain network interactions across cognitive domains remain largely unexplored.

The present study was designed to address this issue and provide a more comprehensive characterization of large-scale network function in human cognition. To this end, we employed a cognitive neuroimaging experiment that taps on multiple key domains in a range of functional complexities, all known to elicit activity from heteromodal inferior parietal lobe regions (Numssen et al. 2021). Specifically, we combined prototypical tasks that exemplify the three domains of attention, semantic cognition, and social cognition with multivariate activity and connectivity analysis approaches (sICA paired with temporal regression and correlational psychophysiological interaction, cPPI) to provide a spatiotemporal characterization of multiple large-scale, overlapping networks that differentially interact across cognitive domains.

A strength of our study design, along with the combination of tasks from three domains into a single experimental run, is the incorporation of different samples of cognitive complexity. Although it remains an open question how to best measure task complexity (Shine and Poldrack 2018), in our experiment, we consider the attention task to be the most simple one, followed by the semantic task, and finally social cognition. This notion is based on the increasing number of cognitive steps necessary to make the task decision for each of these domains. That is, for the invalid condition of interest in the attention task, the participant redirects attention to the unexpected side after the asterisk appears. In the semantic task, the participant must access prior knowledge of vocabulary to make the lexical decision after presentation of a word or pseudoword. In the social cognition task, in order to make a decision, the participant must assume the perspective of a character while following a narrative series of images. For task performance, we consider response time to be a behavioral proxy for domain complexity. For brain function, we employ a recently introduced measure called “functional complexity,” which has been used to quantify the collective dynamics of networks using fMRI connectivity (Zamora-López et al. 2016; Luppi et al. 2021).

Considering whole-brain network overlap, activity, and interactions across these domains should give new insights into how local and distributed resources are flexibly redesignated throughout the cortex during human cognition.

## Materials and Methods

### Participants

We present data from 22 healthy, native German speakers (11 female, mean age 27.9 ± 3.28 years), who took part in the fMRI experiment reported in Numssen et al. (2021). All participants were right handed according to the Edinburgh Handedness Inventory (Oldfield 1971) (laterality quotient > 80%), had normal hearing and normal or corrected-to-normal vision, and no history of neurological or psychiatric disorders or any contraindications against MRI. Participants were recruited from the in-house database at the Max Planck Institute for Human Cognitive and Brain Sciences. All participants gave written informed consent to participate in the experiment approved by the Ethics Committee of the Medical Faculty of the University of Leipzig, Germany (282/16-ek).

### Experimental Design

The fMRI experiment consisted of three sessions in the same healthy volunteers that were performed on separate days, scheduled at least seven days apart to avoid repetition effects. Each session was divided into four runs during which three tasks were administered consecutively in blocks. Within task blocks, trials were presented in an event-related fashion. This design provided us with a large data set that should have sufficient power to detect task-specific activity and connectivity profiles within and between large-scale networks for cognition.

To probe the spatial and temporal characteristics of large-scale task-evoked neural networks, we relied on three tasks that exemplify the larger cognitive domains of attention, semantic cognition, and social cognition. To this end, healthy human volunteers performed a Posner-like attentional reorienting task, a lexical decision task, and a perspective-taking task during fMRI. In previous studies, these tasks have been demonstrated to rely on intact inferior parietal lobe (IPL) function (Rushworth et al. 2001; Binder et al. 2003, 2005). All three tasks followed the rationale of contrasting a target condition that recruits attentional reorienting, semantic processing, or social cognition with one well-matched control condition.

**Figure 1:**
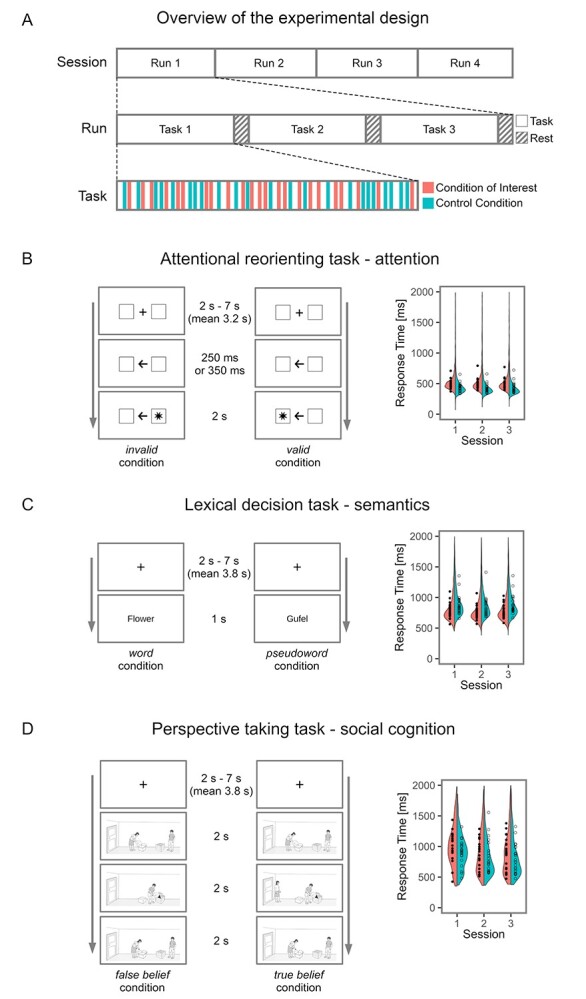
Experimental design and tasks. (*A*) Experimental design. Each of the three sessions consisted of four fMRI runs. In each run, all three tasks were presented in a pseudo-randomized order. All tasks followed the rationale of contrasting a target condition that recruits attentional reorienting, semantic processing, or social cognition with one well-matched control condition. The tasks were presented in an event-related fashion and included rest conditions (16 s) at the end. (*B*–*D*) The tasks on the left, and, on the right of each panel, correct response times are plotted by participant, averaged across trials, for each session and each condition. (*B*) Lexical decision task. Participants performed lexical decisions (word or pseudoword?) on words (target condition) and pseudowords (control condition) based on concrete German nouns and well-matched pseudowords. (*C*) Attentional reorienting task. In each trial, a directional arrow appeared at the center of the screen to direct the subject’s attention to the left or right. In 75% of the trials, the arrow correctly predicted the position of an asterisk (“valid,” control condition); in 20% of the trials, the asterisk appeared on the opposite side and subjects had to reorient their attention (“invalid,” target condition). In 5% of the trials, no response was prompted (catch condition). Subjects indicated the side of the asterisk’s appearance via button press. (*D*) Perspective taking task. In each trial, character A places an object in a container. Thereafter, character B changes the location of the object. Character A has left the room (“false belief,” target condition) or watches the relocation of the object (“true belief,” control condition). Character A then searches for the object at the location congruent with her/his knowledge (expected) or at the contradicting location (unexpected). Participants had to indicate via button press whether character A searched at the expected location or not.

In short, during the attentional reorienting task, a directional arrow appeared at the center of the screen in each trial to indicate, correctly or incorrectly, the position of a visual target presented on the following screen, thereby directing the participant’s attention to the left or right. In the invalid condition, the arrow pointed incorrectly and the target appeared on the opposite side, forcing participants to reorient their attention in the task target condition. Correctly pointed arrows represented the valid control condition. In the lexical decision task, participants performed lexical decisions (word or pseudoword?) about a visually presented word (target condition) or pseudoword (control condition) based on concrete German nouns or well-matched pseudowords. The perspective-taking task required the participant to assume the perspective of one character in a two-character story, presented visually as a three-image comic series consisting of false belief (target condition) and true belief (control condition) scenarios (see Fig. 1, main text). For a detailed description of the tasks, see Numssen et al. (2021). Responses were made via a two-finger button box. Before entering the MRI scanner, the participants underwent task training.

### fMRI Procedure

Data acquisition was performed on a 3 Tesla Siemens Prisma system (Siemens). A whole-brain gradient echo planar (GE-EPI) *T*_2_^*^-sensitive sequence (3 × 3 × 3.2 mm, 0.32 mm gap, 0.5 s TR, 36 slices, 24 ms TE, 45° flip angle) with multiband acceleration was used (Feinberg et al. 2010). Additionally, a high-resolution (1 × 1 × 1 mm voxel size) structural MR image (*T*_1_-weighted) was acquired for each participant using a standard 3D magnetization-prepared rapid acquisition with gradient echo sequence.

### Data Analysis

#### Preprocessing and Univariate Analyses

All fMRI data were preprocessed with a standard pipeline and analyzed with a standard mass-univariate approach as described previously (Numssen et al. 2021). For preprocessing, the raw fMRI data were despiked with 3dDespike from the AFNI toolbox through Nipype v1.5.0 (Gorgolewski et al. 2011). Then, the preprocessing was implemented in a Nipype-based tool, fMRIPrep v1.4.1 (Esteban et al. 2019). The individual *T*_1_ image was intensity-corrected using N4BiasFieldCorrection v2.1.0 (Tustison et al. 2010) and skull-stripped using antsBrainExtraction.sh v2.1.0 with the OASIS (Marcus et al. 2007) template. Brain surfaces were reconstructed using recon-all from FreeSurfer v6.0.1 (Dale et al. 1999). A brain mask was refined to reconcile ANTs-derived and FreeSurfer-derived segmentations of the cortical gray matter in Mindboggle (Klein et al. 2017). Spatial normalization to the ICBM 152 nonlinear asymmetrical template version 2009c (Fonov et al. 2009) was performed through nonlinear registration with the antsRegistration tool 2.1.0 (Avants et al. 2008). Brain tissue segmentation of cerebrospinal fluid, white matter, and gray matter was performed on the brain-extracted *T*_1_ with the FAST tool (FSL v5.0.9 (Zhang et al. 2001)). Functional data were slice–time-corrected with 3dTshift from AFNI v16.2.07 (Cox 1996) and motion-corrected using mcflirt (FSL v5.0.9 (Jenkinson et al. 2002)). Distortion correction was performed with the TOPUP technique (Andersson et al. 2003) using 3dQwarp from the AFNI toolbox. This was followed by coregistration to the *T*_1_ using boundary-based registration (bbregister from FSL v6.0.1; Greve and Fischl 2009) with nine degrees of freedom. Motion correction transformations, field distortion correcting warp, functional-to-anatomical transformation, and T1-to-MNI warp were concatenated and applied in a single step using antsApplyTransforms (ANTs v2.1.0) with Lanczos interpolation. To account for motion-induced artifacts, physiological noise regressors were extracted with the anatomical version of CompCor (Behzadi et al. 2007) (aCompCor). Six components were calculated within the intersection of the subcortical mask and the union of corticospinal fluid and white matter masks. Frame-wise displacement (Power et al. 2014) was calculated using the implementation of Nipype. Statistical Parametric Mapping 12 (SPM 12, Wellcome Department of Imaging Neuroscience) was used to spatially smooth the functional data with a 4-mm full-width half-maximum Gaussian kernel.

#### Spatial Independent Component Analysis

To characterize spatially independent task-active networks in a data-driven manner, a soft parcellation was performed using group spatial independent component analysis (ICA; Calhoun et al. 2004). Preprocessed data were analyzed using the Group ICA for fMRI toolbox (GroupICAT v4.0d), which implements the procedure in four major steps. As a preprocessing step within GIFT, all the time series were intensity-normalized before ICA. First, data dimensions were reduced with a two-step principal component analysis (PCA) procedure, with PCA performed initially at the session level, then concatenated for group-level dimensionality reduction. In the first step in data reduction, the dimensions were reduced from the full time course length (1319 ± 22.5 timepoints, varying across subjects) to 49 per participant and session, which was determined using minimum description length criteria. The second PCA step further reduced the data dimensions from 10 032 total to 49 for the group. The final size in this dimension determined the number of independent components (ICs) extracted using the Infomax algorithm in ICA (Calhoun et al. 2002). To determine the stability of the components, Icasso repeated ICA 50 times (Himberg and Hyvarinen 2003). In a final step, the group-level ICs were back-reconstructed to each session using the GICA method in GIFT. Components were initially scaled to *Z*-scores within each component for the remaining analyses.

#### Network Identification

From the resulting 49 ICs, 30 noise components were identified using visual inspection. In this step, the location of the voxels with the highest *Z*-scores in the component identify the regions with the greatest contribution to the component time course, so components with *Z*-score peaks located outside the cortex, for instance, in the ventricles or on the edge of the brain, were labeled as artifacts (Xu et al. 2015). Of the remaining 19 non-noise components, 11 components of interest were selected. The excluded network components comprised low-level domain-specific networks that are also found during scans without explicit task performance, including ventral visual, medial visual, auditory, motor, and somatosensory networks. The cerebellar and subcortical components were also excluded, leaving the remainder of the analysis to focus on cortical components that perform high-level tasks or contribute to domain-general functions. To characterize the spatial extent of the components at the group level, for each of the 11 selected components, all participant session-level spatial maps were averaged within participant, then submitted to a one-sided *t*-test using a mask of the cerebrum and thresholded at FDR-corrected *P* <0.05, with a minimum of 20 voxels per cluster (AFNI 3dttest++) (Cox et al. 2017). The Eickhoff–Zilles macro label template was used for anatomical labeling (AFNI whereami, CA_N27_ML template). To label the selected components as cortical networks recognized in the literature, the Jaccard similarity coefficients were quantified between pairs from the 11 components and templates of brain networks reported in prior literature (Jackson et al. 2019). The Jaccard index quantifies the similarity of a result and a template through assessing spatial overlap of the two binarized maps (Maitra 2010; Jackson et al. 2019). Template maps were chosen due to their relevance in analytical technique or experimental design. Intensity-based result template maps included 10 ICA-derived networks whose maps were well matched with cognitive profiles in the BrainMap database (Smith et al. 2009), the ALE-derived general semantic cognition network described by Jackson and colleagues (Jackson 2021), and the so-called multiple demand cortex derived from an average of task-based results and reported by Fedorenko and colleagues (Fedorenko et al. 2013; “MDsystem - MRC CBU Imaging Wiki” 2021). Result templates were thresholded to positive voxels with a value of at least 20% of the maximum value in the volume. Additionally, the template battery included binary masks of each network from the 17-network parcellation reported by Yeo and colleagues (Yeo et al. 2011). All template maps were resampled to the resolution of the ICA results before calculating Jaccard similarity indices.

#### Network Activity

Multiple regression analysis was used to assess each IC’s activity during the three tasks. Using SPM12 model setup functions, a GLM design matrix was constructed for each participant’s 12 runs using the FAST(1) algorithm to account for temporal autocorrelation in fMRI data with short repetition times (Corbin et al. 2018). The model included a regressor for the onset and duration of every stimulus in an fMRI run, with six regressors for each task’s target and control conditions, as well as regressors for task instructions, rest periods, attention cues, catch trials, social cognition comics, and wrong trials. Time and dispersion derivatives were included in the model, yielding a total of approximately 759 regressors (varying due to the number of wrong trial regressors per task) for 12 sessions in each subject-specific design matrix. With the three basis functions for each task’s target condition, control condition, and rest period (27 regressors: attention invalid/valid/rest, semantics word/pseudoword/rest, social cognition false belief/true belief/rest, and time and dispersion derivatives of each), the temporal sorting utility in GIFT was used to perform multiple regression analyses between the component time courses and the GLM design matrix at the subject level. The regression step yielded session-specific beta values for each component and each condition that reflect the component’s activity for a given task predictor (11 ICs × 9 conditions × 22 subjects × 12 sessions).

To determine how network activity loaded during each task, pair-wise comparisons of activity estimates were made for all tasks. For each network, primary beta values were averaged across sessions, and GIFT’s “Stats on Beta Weights” utility was used to perform participant-level two-sided *t*-tests. For a general overview of the brain state giving rise to task-relevant network activity, comparisons were made between the target condition and the rest condition for each task (i.e., attention: invalid vs. rest, semantics: word vs. rest, social cognition: false belief vs. rest), and for assessing network activity more specifically, a second set of paired *t*-tests was conducted between each task’s target and control conditions (i.e., attention: invalid vs. valid, semantics: word vs. pseudoword, social cognition: false belief vs. true belief). A significance threshold of *P* <0.05, Bonferroni-corrected for 66 tests (11 ICs × 6 contrasts) was applied.

#### Network Overlap

For each task, components with significant activity during the target condition relative to rest or relative to the control condition were combined to visualize domain-specific network overlap. Thresholded component maps were binarized and added voxel-wise to generate a network participation map reflecting the number of active networks in any given voxel for a task’s target condition. For every task’s target-versus-rest or target-versus-control comparison, the components that loaded positively, or activated, during the tasks were added separately from the components that loaded negatively, or deactivated, during the tasks. To show that the resolved networks are spatially distinct despite their overlap, a spatial correlation of thresholded maps was performed between all network pairs (AFNI 3ddot).

#### Network Interaction

Task-related network interaction was isolated from the component time courses by applying a modified PPI analysis. The correlational PPI analysis followed the procedure described previously by Fornito and colleagues (Fornito et al. 2012). With cPPI analysis, rather than making parameter estimates from a single PPI term derived from a hypothesis-driven selected region of interest, pair-wise interactions are assessed between all regions, or networks, using partial correlations. In the same manner as a traditional PPI analysis, contrasts from conditions of interest are combined with the fMRI time series of regions of interest while controlling for activity of the remaining regions, other stimuli in the task, as well as motion and nonbrain noise. The contrast between the two analyses is that traditional PPI models show effective connectivity based on a hypothesized predictor function whereas cPPI reveals task-specific functional connectivity between all of the networks of interest.

The session-concatenated GLM design matrices, as well as session-concatenated confound matrices constructed from the mean CSF and white matter signals, the global mean, six CompCor, and six motion regressors, were used to carry out cPPI analyses for each participant. On the task level, for every pairwise interaction, two PPI terms were generated with the contrast of interest and the ICA output time series from each of two networks of interest. A partial correlation was performed between the two PPI terms, while controlling for all remaining regressors in the GLM, all noise regressors, and the activity of the nine remaining networks. For assessing task-specific network interaction, contrasts of interest were designed as comparisons between the target and control conditions of each task. Explicitly, that is, invalid versus valid for attention, word versus pseudoword for semantics, and false belief versus true belief for social cognition. Network interaction matrices were computed using all sessions concatenated, resulting in a single symmetric 11 × 11 correlation matrix for each subject, which were then statistically tested at the group level. Interactions were considered significant at *P* < 0.05, Bonferroni-corrected for three tests.

For identifying significant network interactions that occur particular to task performance, partial correlation values were statistically tested as a factor of domain (attention, semantics, social cognition) with analysis of variance (ANOVA) performed at the network interaction level (i.e., between networks, e.g., DMN-to-CON connectivity). For every significant pair-wise network interaction (*P* <0.05, Bonferroni-corrected for 55 tests), three post-hoc *t*-tests were performed pair-wise between all tasks. Significant results from the post-hoc *t*-tests (*P* < 0.05) were visualized in circular graphs plotting the differences between correlation values for each network interaction pair (attention > semantic, attention > social cognition, semantic > attention, semantic > social cognition, social cognition > attention, social cognition > semantic; Fig. 6). To compare overall network interaction across tasks, functional complexity, *C*, was quantified at the participant level, as defined by Zamora-López and colleagues (Zamora-López et al. 2016)

(1) \begin{equation*} C=1-\frac{1}{C_m}{\sum}_{\mu =1}^m\left|{p}_m\left({r}_{ij}\right)-\frac{1}{m}\right|, \end{equation*}

using only the positive cPPI correlations, *r_ij_*, between network pairs (*i, j* = 1,2,...,11) across a range of bin numbers, *m* (10, 15, 20, 25, 30, 35, 40), where |.| is the absolute value, and *p_m_* is the observed distribution of pairwise correlations for a given bin number. Paired *t*-tests of complexity values, averaged over bin numbers for each participant, were performed between tasks. Task response times were used as a behavioral proxy for domain complexity, so paired *t*-tests were also performed between tasks using subject response times, averaged over correct trials within the condition of interest in each task. In this interpretation, slower response time would indicate a more cognitively complex task. Complexity was calculated in MATLAB (The MathWorks, Inc.) and statistical tests were performed in R (R: A language and environment for statistical computing. R Foundation for Statistical Computing, Vienna, Austria. URL https://www.R-project.org/).

## Results

### Independent Components and Network Identification

**Figure 2:**
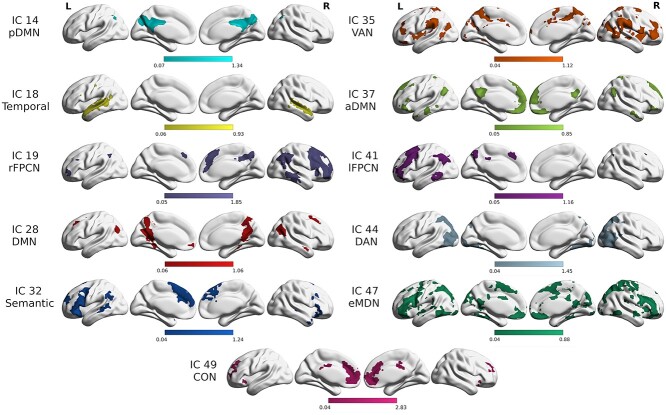
Selected spatial ICA-derived networks. Selected high-order connectivity networks are displayed (one-sided *t*-test results, voxel-level FDR threshold *P* <0.05, 20-voxel cluster minimum). pDMN (IC14, cyan): posterior default mode network, temporal lobe network (IC18, yellow), rFPCN (IC19, lavender): right fronto-parietal control network, DMN (IC28, red): default mode network, semantic network (IC32, blue), VAN (IC35, orange): ventral attention network, aDMN (IC37, yellow-green): anterior default mode network, lFPCN (IC41, purple): left fronto-parietal control network, DAN (IC44, gray-blue): dorsal attention network, eMDN (IC47, green): extended multiple demand network, and CON (IC49, magenta): cingulo-opercular network.

The 11 selected networks of interest, displayed in Figure 2, have spatial topographies that either closely resemble typical high-order intrinsic connectivity networks resolved from resting-state experiments, or they are task-positive networks assumed to emerge specifically for task processes. For this reason, a mix of templates originating from resting-state as well as task-based fMRI studies was used in a labeling procedure based on Jaccard similarity indices between network and template maps. Components were labeled in accordance with the template, or templates, with the highest Jaccard similarity for each network. Networks that are reliably detected most typically in resting-state fMRI experiments, hereafter referenced as named include IC19 as the right frontoparietal control network (rFPCN), IC41 as the left frontoparietal control network (lFPCN), IC44 as the dorsal attention network (DAN), and IC49 as the cingulo-opercular (CON) (Beckmann et al. 2005; Damoiseaux et al. 2006; Smith et al. 2009). In the case of the DMN, four networks were resolved, three of which have been identified previously in resting-state experiments as posterior cingulate/precuneus (pDMN), temporal lobe, and medial-prefrontal (labeled here anterior; aDMN) default mode subnetworks (Zuo et al. 2010), represented by IC14, IC18, and IC37, respectively. The fourth component, IC28, encompasses the major nodes of the classical DMN, such as bilateral angular gyri and anterior and posterior midline structures, and so was assigned the basic DMN label. Supplementary Table 1 includes a descriptive list of the most strongly connected cortical regions in the networks of interest, as well as their highest-matching templates and Jaccard similarity indices.

Given the fMRI data were not acquired when the participants were at rest but when actively participating in three distinct tasks, we also identified task-evoked functional networks that would not emerge merely as resting-state intrinsic connectivity networks (Yeo et al. 2011; Long et al. 2013). The remaining three components (IC32, IC35, and IC47) reflect relatively well-characterized networks that coalesce specifically in particular task-based fMRI experiments. The spatial topography of IC35 matched most closely with Yeo’s 17–network-based template of the ventral attention/salience network A. Thus, IC35 was labeled as the ventral attention network (VAN), which has been characterized using top-down attentional selection tasks as well as seed-based connectivity maps of resting-state data (Fox et al. 2006; Vossel et al. 2014). Covering parts of bilateral ventral frontal cortices and temporoparietal junctions, the VAN is closely situated to, and sometimes overlapping with, the DAN, theoretically allowing an efficient synergy between the two attention systems for fast, successful performance of goal-directed tasks (Spreng et al. 2010; Vossel et al. 2014). Component 47 has the greatest similarity to the template representing the multiple demand network, which is known to aggregate during tasks with multiple cognitive demands (Dosenbach et al. 2006; Duncan 2010; Fedorenko et al. 2013). In most descriptions, the MDN includes bilateral inferior frontal sulci, bilateral anterior insula, and the dorsal anterior cingulate/presupplementary motor area. As it includes additional, stronger contributions in the inferior parietal sulcus to accompany parts of the core MDN regions listed above, IC47 was labeled as the extended multiple demand network (eMDN) (Camilleri et al. 2018). Finally, comprising bilateral regions of inferior frontal gyri (IFG), pars opercularis and pars triangularis, large parts of the inferior parietal lobes (IPL), including angular gyri (AG), and supramarginal gyri, as well as superior parietal lobes (SPL), middle occipital gyri, precuneus, and, notably, left middle temporal gyrus, and bilateral temporal poles, the overall left-lateralized IC32 is identified additionally through Jaccard similarity and labeled as the semantic network. This task-evoked system has an evolving description that has been elucidated by a number of research groups employing different fMRI tasks, lesion studies, and other methods (Binder et al. 2009; Visser et al. 2009; Noonan et al. 2013; Xu et al. 2017; Jackson 2021). For reference, all 49 ICs resulting from the sICA decomposition are displayed in Supplementary Information (Supplementary Fig. 1). For the networks of interest, Supplementary Figure 2 displays the spatial correlations between each network pair, confirming that the networks labeled and used in further analysis are, in fact, spatially distinct from one another.

### Task-Specific Network Activity and Overlap

**Figure 3:**
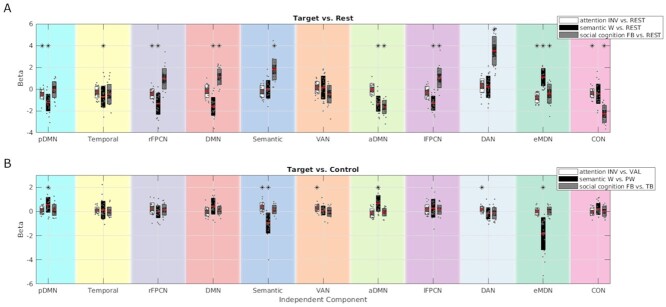
Connectivity networks exhibit domain-specific activity. Plots of the differences between mean beta weights for each target condition against rest (*A*) or control (*B*) baseline from the regression analysis performed on each of the 11 components displayed in Figure 2. White, black, and gray bars represent attention, semantic, and social cognition tasks, respectively. Red line indicates overall mean difference, darker shading within bars indicates standard error of the mean, and the large area of each bar indicates the standard deviation. Individual data points represent the subject-wise difference between the session-averaged betas. Asterisks indicate significance for a given network’s task-wise target versus baseline *t*-test (Bonferroni-corrected *P* <0.05). Error bars show standard error of the mean across subjects. Panel colors correspond to network colors displayed in Figure 2. Attention task: INV = invalid, VAL = valid; semantic task: W = word, PW = pseudoword; social cognition task: FB = false belief condition, TB = true belief condition.

**Figure 4:**
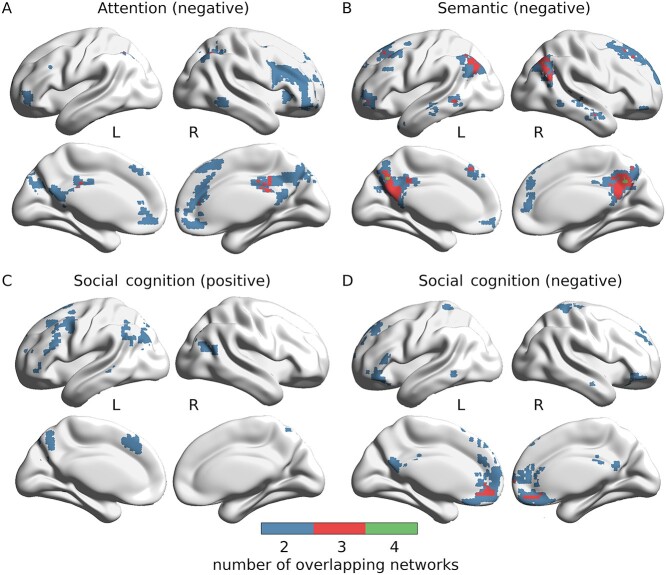
Domain-specific network overlap. Networks with significant domain-specific activity exhibit overlap throughout the cortex, increasing from attention to semantic to social cognition domains. Voxel-wise participation maps are displayed, computed by adding binary masks of all significantly activating or deactivating networks in the target condition versus rest beta comparisons displayed in Figure 3*A*. Overlap maps of deactivating networks are displayed for attention (*A*) and semantic (*B*) domains, whereas social cognition evokes multiple activating (*C*) and deactivating (*D*) networks. Voxel colors indicate the number of overlapping networks in that voxel considered to be active during a specific task, beginning with two. Supplementary Figure 3 shows network overlap for target versus control comparisons.

The task-specific differences between target and rest or control condition betas are plotted by network in Figure 3*A*,*B*, respectively. Supplementary Table 2 reports network-wise task statistics on the beta weights. For each task, networks with significant activity in the rest comparison were combined to identify regions in which multiple task-specific networks overlap (Fig. 4).

Beginning with the attention paradigm, four networks, pDMN, rFPCN, eMDN, and CON, significantly deactivated during the target invalid condition in comparison to rest. Interestingly, the invalid versus valid comparison showed the semantic network, along with DAN and VAN to activate significantly for the more specific condition contrast. The attention overlap map in Figure 4*A* shows that three deactivating networks share space in three large clusters in right AG and SPL, left AG and IPL, and bilateral middle and posterior cingulate cortex. Numerous additional regions have two overlapping deactivated networks, specifically in bilateral parietal, right-biased ventral prefrontal, posterior inferior temporal lobe, and midline cingulate cortices, as illustrated in Figure 4*A*. Supplementary Figure 3*A* displays overlapping regions from the three networks shown to significantly deactivate for the attention target-versus-control comparison, which maximize at two and distribute bilaterally in small clusters in IFG, temporoparietal cortices, and SMA.

For the semantic target-versus-rest comparison, six networks, including all default mode networks (temporal, aDMN, pDMN, and DMN) as well as left and right FPCNs, deactivated during word decisions, while the eMDN activated. When comparing word versus pseudoword conditions, anterior and posterior DMN subsystems activated, while the semantic network significantly deactivated in tandem with the eMDN. Figure 4*B* shows the semantic domain network overlap map for the multiple deactivating networks in the word-versus-rest comparison. A maximum of four deactivating networks overlap in bilateral precuneus and in a small cluster in the right AG. Stepping down to a threshold of three networks extends the posterior medial region of overlap to include a greater portion of bilateral precuneus to middle and posterior cingulate cortices. Three of the six deactivating networks also overlap in five other areas, including bilateral AG, left middle temporal gyrus, and, anteriorly, bilateral middle frontal gyri. When considering regions with at least two overlapping networks with negative activity, the topography includes medial posterior and prefrontal regions, as well as bilateral ventral prefrontal, temporal, and posterior parietal cortices, which together strongly reflect the core topography of the DMN. Notably, in addition to the six deactivating networks that overlap as described, the eMDN significantly activates during the word condition. Supplementary Figure 3*B*,*C* show two networks and their overlapping regions for those found to significantly activate or deactivate, respectively, in the word-versus-pseudoword beta comparison. Overlap of the two activating networks occurs in the bilateral precuneus, posterior and middle cingulate cortices, and angular gyri. The two deactivating networks overlap mostly in large clusters in the left hemisphere that include IFG, superior medial gyrus, precentral gyrus, middle temporal gyrus, temporal pole, SMA, and insula lobe, however overlap also occurs in small clusters in the right IFG, superior medial gyrus, temporal pole, SMA, and insula lobe.

Among the significantly involved networks in the social cognition target-versus-rest comparison were four active ones, including the DMN, semantic network, lFPCN, and DAN, and three deactivating networks, including the aDMN, eMDN, and CON. Perhaps surprisingly, in the more specific task comparison, no network’s activity was significantly different between false belief and true belief conditions. The overlap maps for social cognition-specific activating and deactivating networks are separately displayed in Figure 4*C*,*D*, respectively. Three of the four positively engaged networks overlap in small left hemisphere clusters in AG and middle frontal gyrus, and middle occipital gyrus. Areas in which two social-cognition–positive networks overlap are dispersed throughout the cortex but occur mostly in the left hemisphere, in the middle frontal gyrus, superior medial gyrus, IFG, superior frontal gyrus, precentral gyrus, middle temporal gyrus, AG, IPL, cuneus, and precuneus. Right hemisphere regions where two positive networks overlap include middle temporal gyrus, middle frontal gyrus, middle occipital gyrus, and AG. For negatively active networks in the false belief-versus-rest comparison, all three overlap anteriorly in bilateral rectal and orbital gyri. Two networks deactivated in the task in regions of the brain carrying an anterior bias, touching in the bilateral superior medial gyri, superior frontal gyri, IFG, and anterior cingulate cortices but also including parts of bilateral middle temporal gyri, precentral gyri, SPL, precuneus, and posterior cingulate cortices.

### Network Interaction

**Figure 5:**
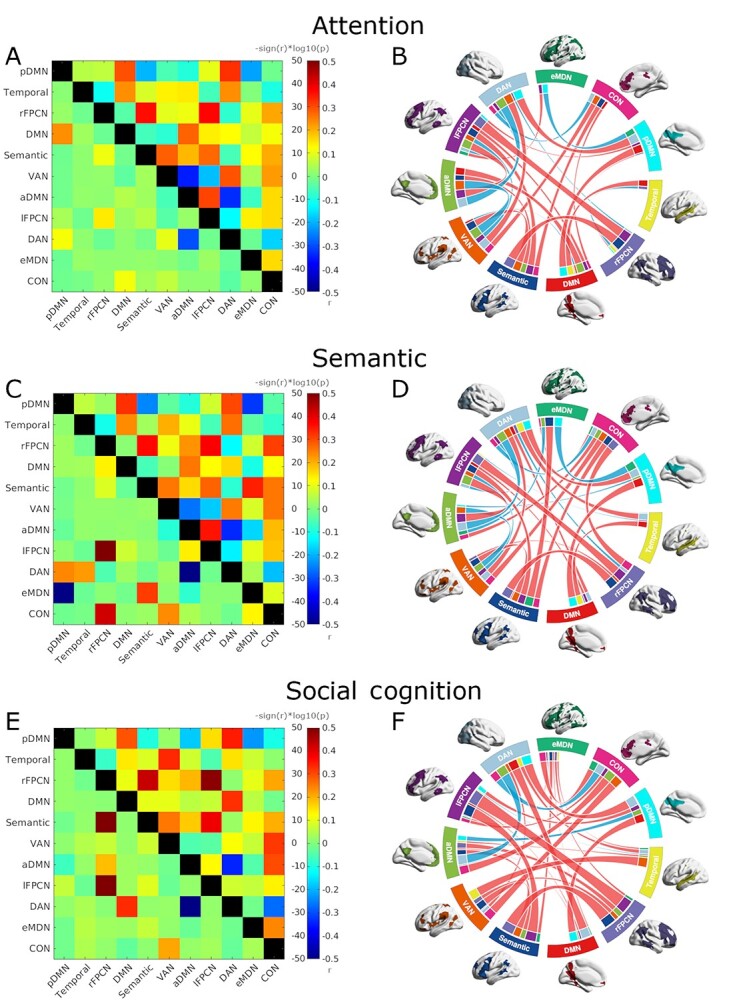
Network interaction by cognitive domain. Partial correlations (*r*) from the cPPI analyses for task-specific contrasts (target vs. control condition) are plotted as matrices and circular graphs for attention (*A* and *B*, respectively), semantic (*C* and *D*, respectively), and social cognition (*E* and *F*, respectively) domains. Results are scaled in the lower triangles of the matrices as –sign (*r*) ^*^ log10 (*P*-value), which displays the strength of the correlation in combination with the statistical significance, thus emphasizing the most stable interactions. The circular graphs are thresholded using mean *P*-values across participants at *P* < 0.05, Bonferroni-corrected for three tests. In the circular graphs, line colors indicate whether the interaction is positive (red) or negative (blue), whereas line thickness indicates the strength of the interaction. Line thickness is scaled within each plot. Network colors and labels correspond to Figure 2. cPPI correlation values and statistics are detailed in Table 1.

The cPPI analysis was applied to the data to determine how the networks of interest interact across cognitive domains, and it resulted in correlation matrices for each cognitive domain, with interactions quantified between each network pair. Figure 5 displays domain-specific network interaction as matrices (left column), providing insight into interaction strength, and circular graphs (right column), allowing an easy view of positive and negative interactions between the spatially distributed networks. Interaction values and statistics are detailed by network in Table 1. The network interaction plots portray a considerable number of significant task-specific interactions between the queried networks. In fact, all 11 networks had at least two significant interactions in each task. First, we consider network interaction by cognitive domain. Thereafter, we present network interaction compared across domains. Supplementary Figure 4 plots participant-level network interactions by task to give an impression of variability across subjects and network pairs for each domain.

For attention, network interaction is depicted in the top row of Figure 5*A*,*B*. In total, 30 pair-wise interactions were significant in the cPPI calculations, 11 of which were negative, with notable characteristics described as follows. Strong positive interactions occurred between the two lateralized FPCNs. Of all the networks included in the cPPI analysis, the lFPCN showed the most (eight) significant interactions for attention. The dorsal attention, ventral attention, and default mode networks also showed their strong roles in the attention task with six significant interactions each. The dorsal and ventral attention networks were correlated in the attention task, even while each had negative and positive interactions, respectively, with CON. Both DAN and VAN were anticorrelated with the aDMN subnetwork, and there was also negative interaction between the eMDN and the pDMN, reflecting typical interactions between networks considered task positive and task negative in the literature (Fox et al. 2005).

Network interaction is plotted for the semantic domain in the second row of Figure 5, and 33 pair-wise interactions, including 12 negative interactions, were significant in the cPPI calculations as thresholded in Figure 5*D*. Some network features that persisted from attention to semantics were the significant positive interaction between the two lateralized frontoparietal control networks, as well as anticorrelations between the eMDN and pDMN, and between both attention networks and the aDMN. Also like in attention, while correlated with each other, the DAN and VAN had negative and positive interactions, respectively, with the CON. With eight significant interactions, the DAN appears to be a prominent network for lexical decision making. The lFPCN again showed prominence in the task with seven significant interactions. Perhaps demonstrating the increased complexity of the semantic domain over attention, four networks had six significant interactions in the task, including the semantic, ventral attention, cingulo-opercular, and anterior default mode networks.

The bottom row in Figure 5 displays the network interaction for social cognition, and Figure 5*F* shows the 34 significant interactions, according to cPPI calculations. Overall, social cognition had more significant interactions compared to attention and semantics, however, relatively more of these interactions were comparably weak, and only seven were negative. Despite having a greater number of weak interactions between networks than in the other two tasks, social cognition maintained strong positive coupling between the two frontoparietal control networks, as well as anticorrelations between the DAN and aDMN. Another set of network interactions stayed similar across domains, that is, the DAN anticorrelated with the CON, VAN correlated with the CON, and the two attention networks positively interacted with each other. For social cognition, the lFPCN once again appears to be a critical network, along with the aDMN, each with eight significant interactions in the task. The eMDN plays a prominent role, as well, with seven significant interactions in the domain. Notably for social cognition, the DAN has a strong interaction with the DMN, as well as the pDMN.

### Network Interaction across Tasks

**Figure 6:**
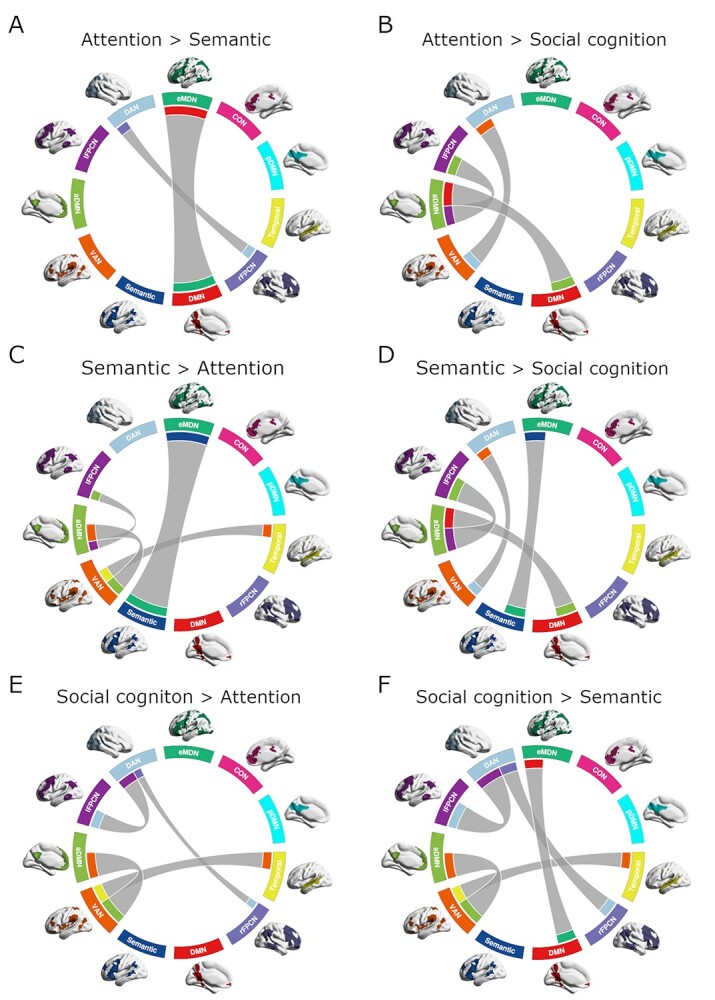
Between domain network interaction differences. Differences between cPPI correlations across cognitive domains for (*A*) attention > semantic, (*B*) attention > social cognition, (*C*) semantic > attention, (*D*) semantic > social cognition, (*E*) social cognition > attention, and (*F*) social cognition > semantic. Line thickness indicates the network interaction difference between the compared tasks and is scaled within each plot. Network colors correspond to the assignments in Figure 2. Interactions found significant in the ANOVA are plotted (one-factor ANOVA, Bonferroni-corrected *P* <0.05; *P* <0.05 for post-hoc *t*-tests). cPPI correlation values and statistics are detailed in Table 1.

Comparing interactions across the complexity-varied tasks gives insight into potentially critical domain-specific network characteristics. Figure 6 depicts the differences in interaction between task pairs, allowing to infer which network pairs were more synchronized in one task relative to another. The ANOVA performed on the cPPI correlation values determined whether each interaction was significant in the analysis (see Table 1). Figure 6 is arranged such that the rows, top to bottom, display domain-specific results in order of the tasks’ progressively increasing cognitive complexity. The first row in Figure 6 shows interactions that were significantly greater in the attention task than in the semantic (Fig. 6*A*) and social cognition (Fig. 6*B*) tasks. The middle row features plots with interactions that were stronger in the semantic task than in the attention (Fig. 6*C*) and social cognition (Fig. 6*D*) tasks. Finally, the third row shows interactions that were stronger for social cognition than attention (Fig. 6*E*) and semantics (Fig. 6*F*). Already, it appears that the number of significantly stronger network interactions increases correspondingly with domain complexity. The attention comparison against semantics showed two interactions to be greater, between the eMDN and DMN and between the DAN and rFPCN. Against social cognition, three interactions appeared stronger for attention, including the DAN with VAN, and aDMN with both DMN and lFPCN. Reversing the comparisons, semantics and social cognition both had four interactions that were significantly stronger than in attention, two of which were common differences, that is, the VAN’s interaction with the temporal lobe and anterior default mode networks. The remaining two pairs of networks that interacted more in semantics than attention were the eMDN with the semantic network, and aDMN with lFPCN. The remaining two pairs of networks that interacted more in social cognition than attention were the DAN with the left and right FPCNs. Compared to social cognition, semantics showed four more strongly coupled networks, most notably the eMDN to the semantic network. The additional stronger network pairings are the same as those found significant in the comparison of attention against social cognition, namely the DAN with VAN, and the aDMN with DMN and lFPCN. Finally, social cognition showed five stronger network interactions compared with semantics, including the DAN with the left and right FPCNs, and the VAN with temporal lobe and anterior default mode networks, four stronger interactions in common with the comparison against attention, as well as the eMDN with the DMN. An immediately visible pattern of network connectivity common across tasks is the persistent inclusion of at least one of the two FPCNs, which had significantly greater domain-specific interaction with at least one other network. Other prominent networks across tasks include the eMDN, DAN, VAN, aDMN, and DMN whereas the temporal lobe and semantic networks appear to have more task-specific coupling.

**Table 1:** Network interaction statistics

Network	Inter- acting Network	Attention invalid versus valid			Semantic word versus pseudoword			Social cognition false belief vs. true belief			ANOVA		Post-hoc t-test		
											p (d.o.f. = 2)	F	Attention versus Semantic	Semantic versus Social cognition	Social cognition versus. Attention
		r	SD	p	r	SD	p	r	SD	p			p	p	p
pDMN	Temp	0.053	0.125	7.00E-02	0.062	0.139	2.91E-02	0.013	0.164	1.68E-02	0.489	0.72			
	rFPCN	0.060	0.153	**9.63E-03**	0.025	0.142	**1.24E-03**	0.070	0.143	2.35E-02	0.567	0.57			
	DMN	0.294	0.167	**4.08E-24**	0.328	0.181	**1.70E-03**	0.289	0.148	**1.55E-02**	0.708	0.35			
	Sem	-0.196	0.138	2.41E-02	-0.244	0.141	3.75E-02	-0.094	0.148	4.14E-02	3.20E-03	6.30			
	VAN	-0.054	0.168	6.27E-02	-0.034	0.149	7.17E-02	0.021	0.179	3.44E-02	0.303	1.22			
	aDMN	-0.092	0.191	6.43E-02	-0.091	0.187	**3.08E-03**	-0.177	0.200	**3.57E-07**	0.245	1.44			
	lFPCN	0.099	0.117	**1.38E-04**	0.076	0.109	4.71E-02	0.156	0.173	**4.75E-07**	0.141	2.02			
	DAN	0.324	0.200	**3.11E-12**	0.304	0.203	**1.16E-24**	0.346	0.194	**1.56E-02**	0.783	0.25			
	eMDN	-0.217	0.129	**5.79E-03**	-0.321	0.115	**4.97E-53**	-0.229	0.131	3.63E-02	0.014	4.60			
	CON	-0.017	0.151	2.52E-02	-0.040	0.170	2.64E-02	-0.103	0.180	4.15E-02	0.217	1.57			
Temp	rFPCN	-0.115	0.133	**1.54E-02**	-0.108	0.154	5.84E-02	-0.072	0.164	4.50E-02	0.600	0.52			
	DMN	0.231	0.139	**8.58E-04**	0.237	0.167	**4.86E-04**	0.141	0.154	5.97E-02	0.078	2.66			
	Sem	0.085	0.159	4.53E-02	0.033	0.179	4.48E-02	0.086	0.173	2.44E-02	0.499	0.70			
	VAN	0.134	0.168	4.41E-02	0.200	0.147	1.71E-02	0.335	0.192	**1.44E-05**	**7.91E-04**	8.02	**2.05E-03**	**2.99E-05**	**1.82E-06**
	aDMN	0.141	0.145	1.76E-02	0.103	0.173	2.08E-02	0.076	0.192	**1.07E-02**	0.449	0.81			
	lFPCN	-0.059	0.170	1.74E-02	-0.058	0.192	**6.54E-03**	-0.049	0.215	**1.11E-02**	0.981	0.02			
	DAN	0.231	0.161	4.33E-02	0.264	0.184	**1.32E-22**	0.152	0.201	**1.08E-04**	0.117	2.22			
	eMDN	0.001	0.215	**8.24E-03**	-0.037	0.198	**8.10E-03**	0.072	0.210	**3.80E-06**	0.217	1.56			
	CON	-0.100	0.172	6.82E-02	-0.075	0.162	8.31E-02	0.015	0.181	**4.30E-05**	0.073	2.73			
rFPCN	DMN	-0.039	0.189	**8.52E-03**	0.011	0.178	**2.46E-14**	0.104	0.167	7.43E-02	0.032	3.64			
	Sem	0.362	0.143	**1.92E-11**	0.364	0.136	**1.60E-09**	0.429	0.134	**1.89E-144**	0.192	1.70			
	VAN	0.096	0.193	2.68E-02	0.107	0.211	**1.58E-02**	0.154	0.236	**1.32E-07**	0.632	0.46			
	aDMN	0.108	0.179	5.38E-02	0.227	0.174	4.50E-02	0.192	0.169	**2.31E-19**	0.073	2.73			
	lFPCN	0.368	0.141	**8.08E-15**	0.377	0.122	**5.04E-69**	0.488	0.148	**4.24E-142**	8.06E-03	5.21			
	DAN	-0.080	0.110	**6.73E-03**	-0.128	0.083	**1.10E-05**	0.013	0.114	7.18E-02	**1.07E-04**	10.61	**3.81E-02**	**1.11E-05**	**0.001**
	eMDN	0.098	0.134	4.15E-02	0.077	0.138	7.01E-02	0.098	0.195	**7.79E-04**	0.883	0.12			
	CON	0.210	0.215	2.22E-02	0.312	0.179	**1.18E-42**	0.210	0.229	1.71E-02	0.182	1.75			
DMN	Sem	-0.061	0.202	5.41E-02	-0.056	0.180	**2.24E-03**	0.100	0.166	4.08E-02	6.49E-03	5.46			
	VAN	-0.084	0.245	**3.87E-04**	0.006	0.224	4.79E-02	0.098	0.219	2.27E-02	0.038	3.43			
	aDMN	0.275	0.133	**1.28E-05**	0.256	0.171	2.11E-02	0.056	0.169	**1.08E-03**	**2.01E-05**	12.90	0.404	**1.75E-06**	**4.69E-07**
	lFPCN	0.139	0.137	**3.88E-03**	0.121	0.143	**4.14E-08**	0.068	0.138	**9.90E-04**	0.219	1.56			
	DAN	0.122	0.231	2.83E-02	0.161	0.210	**3.74E-03**	0.326	0.156	**9.98E-34**	3.07E-03	6.35			
	eMDN	0.065	0.136	3.95E-02	-0.102	0.154	3.33E-02	0.055	0.160	**3.31E-03**	**4.99E-04**	8.60	**1.15E-06**	**7.09E-04**	0.794
	CON	0.114	0.190	**1.03E-11**	0.118	0.168	4.02E-02	-0.014	0.205	**1.03E-03**	0.036	3.50			
Sem	VAN	0.280	0.194	**5.12E-04**	0.257	0.208	**8.08E-03**	0.256	0.206	**4.58E-08**	0.905	0.10			
	aDMN	0.193	0.131	**1.38E-03**	0.157	0.158	7.49E-02	0.175	0.154	**7.78E-03**	0.718	0.33			
	lFPCN	0.273	0.119	**6.69E-06**	0.277	0.110	**5.19E-03**	0.388	0.143	**9.96E-10**	4.10E-03	6.01			
	DAN	-0.058	0.140	8.43E-02	-0.084	0.123	5.59E-02	0.024	0.164	2.25E-02	0.038	3.45			
	eMDN	0.122	0.169	2.37E-02	0.340	0.136	**3.06E-32**	0.132	0.197	**2.52E-07**	**4.98E-05**	11.64	**3.20E-06**	**3.23E-05**	0.751
	CON	0.205	0.183	**5.94E-04**	0.254	0.204	**3.06E-03**	0.203	0.185	4.45E-02	0.608	0.50			
VAN	aDMN	-0.350	0.213	**1.79E-06**	-0.263	0.238	**1.93E-09**	-0.051	0.207	4.45E-02	**9.44E-05**	10.78	**6.23E-05**	**2.47E-06**	**3.66E-09**
	lFPCN	-0.190	0.220	**5.35E-05**	-0.177	0.215	**1.07E-02**	-0.036	0.236	**7.85E-03**	0.048	3.19			
	DAN	0.290	0.119	**9.31E-11**	0.252	0.134	**2.11E-03**	0.120	0.166	**5.64E-03**	**4.20E-04**	8.82	0.103	**3.39E-04**	**2.94E-08**
	eMDN	0.039	0.135	8.36E-02	0.085	0.136	4.48E-02	0.068	0.185	1.88E-02	0.602	0.51			
	CON	0.219	0.206	**8.49E-07**	0.257	0.173	**1.73E-24**	0.318	0.151	**1.95E-21**	0.188	1.72			
aDMN	lFPCN	0.308	0.154	**9.38E-03**	0.355	0.143	**1.02E-14**	0.128	0.217	2.11E-02	**1.30E-04**	10.35	**0.007**	**4.20E-07**	**5.90E-05**
	DAN	-0.335	0.105	**8.81E-31**	-0.338	0.075	**6.92E-135**	-0.341	0.117	**1.74E-54**	0.979	0.02			
	eMDN	-0.068	0.201	6.02E-02	-0.166	0.213	**1.13E-08**	-0.043	0.225	**6.32E-03**	0.140	2.03			
	CON	0.157	0.169	4.61E-02	0.189	0.193	**5.98E-05**	0.300	0.171	**9.80E-03**	0.026	3.87			
lFPCN	DAN	-0.129	0.192	**1.52E-02**	-0.140	0.161	**1.53E-02**	0.077	0.201	2.54E-02	**2.40E-04**	9.54	0.581	**2.20E-07**	**1.90E-07**
	eMDN	0.137	0.156	**2.48E-03**	0.094	0.143	9.02E-02	0.063	0.218	**8.99E-03**	0.379	0.98			
	CON	0.159	0.162	2.83E-02	0.182	0.179	4.06E-02	0.133	0.205	**6.48E-04**	0.673	0.40			
DAN	eMDN	-0.023	0.147	**2.88E-03**	0.043	0.145	7.00E-02	-0.015	0.180	2.65E-02	0.324	1.15			
	CON	-0.169	0.123	**1.59E-04**	-0.190	0.129	**1.47E-02**	-0.271	0.128	**1.95E-03**	0.023	4.02			
eMDN	CON	0.159	0.175	3.89E-02	0.092	0.188	**5.97E-14**	0.239	0.151	**1.27E-04**	0.023	4.01			

Mean partial correlations from cPPI analysis. Bold text indicates significant interactions from cPPI *P*-values (left side, Bonferroni-corrected *P* <0.05 for three tests), in the ANOVA testing network interactions across tasks (right side, Bonferroni-corrected *P* <0.05 for 55 tests), and in the post-hoc *t*-tests (*P* <0.05); SD, standard deviation; *P*, *P*-value; *F*, *F*-statistic; d.o.f., degrees of freedom.

**Figure 7:**
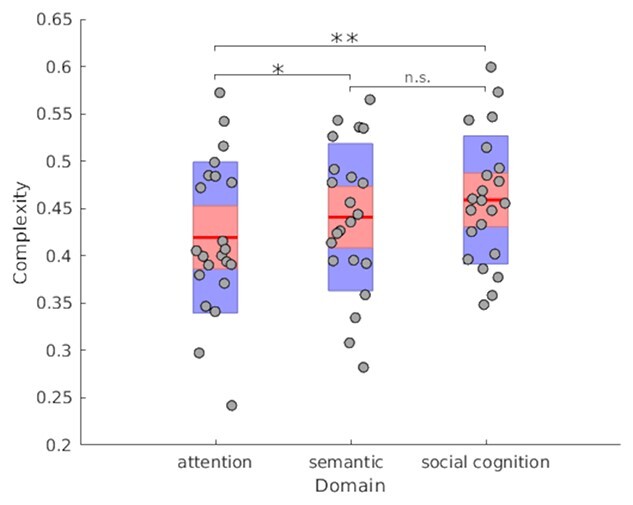
Network complexity across domains. Complexity values are plotted by cognitive domain, with each data point representing average complexity within each subject. Red lines are the mean complexity across subjects for each domain. Light red regions show 95% confidence intervals and blue regions indicate one standard deviation of the mean. ^*^P <0.05, ^*^^*^P <0.01, n.s.: Not significant.

Network interactions were summarized for each domain using calculations of complexity derived from each subject’s cPPI matrix. Figure 7 shows the mean complexity for each subject plotted by domain. Paired *t*-tests between attention and semantics showed semantics to be significantly more complex than attention (*t* = −2.3715, df = 21, *P* = 0.01368), and, whereas the mean complexity of social cognition (0.459 ± 0.0662) was greater than that of semantics (0.4409 ± 0.0759), the difference was not significant (*t* = −1.208, df = 21, *P* = 0.1202). On the other hand, social cognition was, logically, significantly more complex than attention (*t* = −3.2098, df = 21, *P* = 0.002103). Paired *t*-tests performed on mean response times as a behavioral proxy for task complexity showed social cognition to be significantly slower than attention (*t* = −10.202, df = 21, *P* = 6.819e−10), and semantics (*t* = −3.39, df = 21, *P* = 0.001381), and semantics to be significantly slower than attention (*t* = −20.587, df = 21, *P* = 1.051e−15).

## Discussion

Efficient human cognition requires flexible interaction between distributed networks in the brain for different specialized functions. Although classical approaches to understand brain function supporting cognition are generally segregated into isolated studies within key domains like attention, language, and social cognition (Shine and Poldrack 2018), we bridged these domains in a comprehensive fMRI study and characterized 11 large-scale, overlapping spatiotemporal networks across tasks. As a key finding, we demonstrate that higher-order functional network connectivity increases with increasing cognitive complexity across the three domains. In line with recent multitask fMRI studies (Cole et al. 2014; Krienen et al. 2014), we show a common functional structure across tasks. Extending previous work, we further present specific network activity and interactions that dissociate each domain. Additionally, by employing three tasks known to activate regions within the commonly designated task-negative default mode network, we demonstrate that canonical intrinsic connectivity networks fractionate into subsystems as well as aggregate as task-evoked networks that interact distinctly to perform tasks (Andrews-Hanna et al. 2010; Vatansever et al. 2015a; Buckner and DiNicola 2019). By elucidating domain-specific activity and interactions of multiple spatially overlapping and interdigitated cortical networks, our results support a holistic and dynamic perspective of flexible network reconfiguration and integration for diverse interactive cognitive functions.

### Network Activity and Interaction Increase in Complexity with Cognitive Domain

We demonstrate that increasing complexity in cognitive domains is paralleled by increased complexity in brain function. This was achieved by resolving spatially independent networks, inspecting their temporal behavior in the context of each task, and ultimately, quantifying the complexity of network interaction for each domain. In previous work, graph analyses have revealed that global brain modularity decreases during tasks. More specifically, as tasks become more complex, patterns of connectivity between networks show less segregation and whole-brain integration increases (Mattar et al. 2015; Vatansever et al. 2015b; Cohen and D’Esposito 2016; Hearne et al. 2017). Our results transfer this observation across highly complex cognitive domains, including semantic processing and social cognition.

We identified 11 cohesive high-order large-scale networks and used multiple regression and cPPI analyses to quantify their domain-specific activity and interactions. Our results show that network involvement and connectivity increased across cognitive domains, from attention to semantics to social cognition. Likewise, using response times as a behavioral measure of task complexity, trial performance was significantly slower from attention to semantics to social cognition. Statistics on network activity in the target-versus-rest and target-versus-control comparisons showed significant contributions from four deactivating and three activating networks, respectively, for attention. Semantic target comparisons showed one activating and six deactivating networks as significant against rest, and two of each activating and deactivating networks significant against the control. Finally, the target-versus-rest activity comparison for social cognition had the highest number of significantly active networks, four positive and three negative, relative to the other two tasks. Nonetheless, the more specific target-control comparison showed only small differences in activity across all networks. This may seem paradoxical, but although no whole network’s activity is significant, regions, or nodes, within a network may also be activating and deactivating, thus masking subtle or granular activity changes on the whole network level while still affecting network connectivity and interactions. Furthermore, when evaluating the comparisons between target and control conditions for all tasks, one may consider that the false-belief and true-belief conditions present the most similar trials within a task. As a contrasting example, the control condition for the lexical decision task is a pseudoword, so a relevant network like the semantic network is not expected to activate in control trials.

Examining task-modulated connectivity across domains, recent work by Di and Biswal emphasizes the value of taking into account whole-brain connectivity in task experiments in addition to the attention given to regions of task-evoked activity (Di and Biswal 2019). In our results, when considering interaction between networks (Fig. 6), there is a net number of one significantly greater interaction for social cognition compared to each attention (social cognition > attention: 4, attention > social cognition: 3) and semantics (social cognition > semantics: 5, semantics > social cognition: 4). Despite the fact that many of the internetwork connections were weaker for social cognition, this result possibly reflects the aforementioned potential within-network heterogeneity. Meanwhile, the semantic domain showed two more interactions that were significantly stronger against attention (semantics > attention: 4, attention > semantics: 2). Following suit, comparing complexity across tasks showed network interaction during semantics and social cognition to be significantly more complex than for the attention domain. Although mean complexity for social cognition was greater than that of semantics, the lack of significant difference is perhaps more specifically untangled in the network interaction comparisons between tasks, with semantics having two interactions greater than in attention versus one for social cognition against attention. This result may also speak to the similarities between semantic and social cognition domains and the fact that the social cognition task might call upon some semantic functions (Binder and Desai 2011; see also Bzdok, Varoquaux, et al. 2016). The complex network interaction results for social cognition corroborate Theory of Mind meta-analyses that show adaptations of a core network with specific cross-network integration patterns that depend on the aspect of social cognition being probed (Schurz et al. 2014, 2020). Thus, overall, the quantified complexity of each cognitive domain is reflected in the network activity as well as interactions between multiple brain networks during task performance.

### A Common Core Network Structure across Domains Differentially Reconfigures for Task Performance within Each Domain

Prior neuroimaging studies have demonstrated high similarity in the functional architecture of the brain during rest and task states; nevertheless, the subtle task-dependent reconfigurations that emerge from the basic functional scaffold are critical (Cole et al. 2014; Krienen et al. 2014; Cohen and D’Esposito 2016). Furthermore, task-specific adaptations to connectivity are more likely to occur on the basis of functional communities, that is, cohesive network couplings or coactivations, rather than recruitment of independent brain regions (Krienen et al. 2014; Mattar et al. 2015). In line with this research, temporal analyses of the 11 high-order networks revealed motifs of shared structure across tasks, as well as distinct features that dissociated the cognitive domains. The common cross-domain structure is perhaps most apparent from the network interaction differences between tasks (Fig. 6). Overall, the aDMN, lFPCN, DAN, and VAN played prominent roles across domains. Each of these four networks had a significantly greater interaction with another network in five of the six between-domain interaction comparisons. In addition, the eMDN and DMN each had a significantly stronger correlation with another network in four of the six comparisons. Because it is likely to play a crucial role in executive function across tasks, the eMDN highlights domain-specific contributions of networks through its significant interactions. For instance, compared with attention and social cognition, network interaction for semantics showed significantly higher correlation between the eMDN and the semantic network. For the most part, previous studies comparing task- and rest-associated network architectures do not explicitly investigate the role of MDN as a core network, perhaps historically because it does not appear as a canonical intrinsic connectivity network. However, there is growing attention to the role of the eMDN as part of the brain’s “functional core” (Dosenbach et al. 2006; Duncan 2010; Fedorenko et al. 2013; Camilleri et al. 2018; Diachek et al. 2020), to which the findings of this study contribute support. Evidence from recent studies dissociate contributions of the MDN from those of domain-specific networks such as the language network or theory of mind network in naturalistic stimulus conditions, emphasizing the domain-general nature of the MDN and its role in overall cognitive control (Paunov et al. 2019; Wehbe et al. 2021). Likewise, the results of this study show contributions from the eMDN to accompany activity of task-specific networks as captured most clearly through the cross-domain interaction comparisons. Together with the lFPCN, DAN, and VAN, the eMDN includes regions demonstrated as hubs that actively switch between networks across tasks (Cole et al. 2013; Cocuzza et al. 2020), which is in line with our findings showing high cross-network interactions and spatial overlap among the networks.

In addition to domain-specific eMDN coupling, our results further align with the concept of an overarching set of network coactivation patterns that specifically modify during cognition (Krienen et al. 2014; Bzdok, Varoquaux, et al. 2016). In all three domains, the two bilaterally mirrored FPCNs consistently correlated with each other and widely interacted with other networks. Moreover, at least one of the two FPCNs had a significantly stronger task-specific interaction with another network for all cross-task comparisons. Amidst an increasing understanding of the topography and function of various large-scale networks that include frontal and parietal regions (Dosenbach et al. 2008; Duncan 2010; Menon and Uddin 2010; Fedorenko et al. 2013; Crittenden et al. 2016; Camilleri et al. 2018), the laterally situated FPCNs distinguish themselves in their roles as functional hubs and integrators between other networks. Therefore, both FPCNs are often considered together as a single dynamic FPCN (Crittenden et al. 2016; Dixon et al. 2018; Marek and Dosenbach 2018; Cocuzza et al. 2020). This comparative uniformity is reflected in their tight coupling and similar, but not identical, interactions across tasks.

In concert with the FPCN coupling, the common functional motif is evident from other consistently correlated or anticorrelated pairs of networks across domains. Across domains, the task-positive DAN was anticorrelated with the aDMN, but also correlated with the pDMN. Additionally, CON sustained negative and positive interactions with the DAN and VAN, respectively, whereas the two networks remained correlated with each other throughout the tasks. Although classical functional connectivity literature emphasizes a generally persistent anticorrelation between the DMN and putative task-positive networks (Fox et al. 2005), deeper investigations into relationships between networks show that network coupling, as well as anticoupling, is not so straightforward (Chen et al. 2013; Cocchi et al. 2013; Dixon et al. 2017; Gordon et al. 2020). In fact, recent work measuring regional brain metabolism in relation to positive and negative fMRI responses through contrasts of working memory and rest periods demonstrated that regions exhibiting decreases in fMRI signal do not necessarily show a drop in glucose energy consumption (Stiernman et al. 2021). This was most prominently the case for the temporal lobes and anterior medial prefrontal cortex, which may capture functional specificity that separates these regions into default mode subnetworks, which will further be addressed in the Fractionating and Aggregating Networks section. Overall, the results of the study showed heterogenous metabolism throughout the DMN, highlighting the error in assuming that “task-negative” networks actually reduce their activity when the fMRI response is measured to decrease in an experiment.

Upon this scaffold of supportive networks, specific between-network interactions dissociate each cognitive domain in our data. Attentional reorientation is known to engage both ventral and dorsal attention networks, whose interactions are posited to facilitate a combination of top-down and bottom-up processes (Corbetta et al. 2008). Attention-demanding tasks have been used to demonstrate strong anticorrelation between task-negative DMN and task-positive networks (Fox et al. 2005). Our results are in agreement with these findings, showing correlation between the DAN and VAN and anticorrelation between DAN and aDMN in the attention domain. The cross-domain comparisons highlight some of these relationships. In particular, compared with social cognition, attention shows greater interaction between the DAN and VAN. Also, a stronger relationship between the DAN and the rFPCN for attention relative to semantics likely reflects increased attentional and general cognitive control demands during the attentional reorienting condition. Likewise, against attention, both social cognition and semantics have greater connectivity between VAN and the temporal lobe and anterior default mode subnetworks, which, from the perspective of attention, would mean a greater anticorrelation between that domain’s task-positive and task-negative networks.

For the semantic domain, the most explicit network-based dissociation from the other two domains is the activity and interaction of the semantic network. The specific target-versus-control test on activity betas showed significant deactivation in that network only in the semantic task. Accompanying the semantic network was significant eMDN deactivation, which translates in the network interaction computations to strong interaction between the two networks that is significantly greater in the semantic domain than attention and social cognition. This result is supported by a recent study specifically examining the distinction and relationship between the language network and the multiple demand system that claims the MDN’s role during sentence comprehension is not specific to language demands but merely serves to facilitate task function (Diachek et al. 2020). The semantic network has been extensively characterized in the literature, and its distinct spatial coherence, as well as its characteristic deactivation, agrees with our results (Binder et al. 2009; Binder and Desai 2011). As previously mentioned, network deactivation does not necessarily mean a decrease in energy consumption, and it is noteworthy that we find domain-specific deactivation of the semantic network with eMDN in the target-versus-control comparison (Stiernman et al. 2021). In our study, the observed deactivation likely reflects less task demands for word relative to pseudoword decisions (e.g., Jefferies 2013; Humphreys et al. 2015; Kuhnke et al. 2021). The cross-domain interaction comparisons show that, aside from the eMDN-semantic network coupling, interactions between the lFPCN and aDMN and between the DAN and VAN are greater for both attention and semantics relative to social cognition. If the aDMN is to be considered as one of the core functional networks in these domains, then the increased interaction between lFPCN and aDMN, a correlation that was also significantly greater in semantics than attention, may reflect the specific processes of lFPCN supporting language function in the task. This notion would be consistent with an fMRI experiment contrasting speech production against counting or simple decision-making (Geranmayeh et al. 2014). The other significant increases for aDMN interactions in semantics compared to attention are in common with social cognition over both attention and semantics, that is, the increased coordination of the task-positive VAN with aDMN and temporal lobe networks, likely reflecting the support of general semantic processes, which may overlap to some degree for both domains (Bzdok, Hartwigsen, et al. 2016).

As discussed here, one distinctive network feature of social cognition is an overall greater number of significant comparisons for network activity and interaction than attention and semantics. Examining the cross-domain comparisons of network interaction, greater connectivity occurs between the DAN and the left and right FPCNs for social cognition than the other two domains, a relationship that appears specific to this domain. That is, social cognition additionally exhibits closer coupling of the VAN with temporal lobe and anterior default mode networks compared to attention and semantics, although this motif is also demonstrated in the semantics greater than attention comparison. While the FPCNs and DAN show a particular interrelationship for social cognition, another interesting feature highlighted in the cross-domain comparisons is the stronger interaction between the eMDN and DMN for social cognition relative to semantics. Although we labeled no specific Theory of Mind network, considering its previously mentioned overlap with DMN (Schurz et al. 2014), the relatively increased interaction between eMDN and the DMN perhaps reflects the core task-supporting network’s context-specific coupling for the social cognition domain. Accordingly, Schurz et al. (2020) also suggest an increase in network integration with increasing amounts of effort and control in cognition, which may be reflected in the greater number of significant interactions found for social cognition in our study, a smaller proportion of which were negative. Our results complement and extend these findings by showing that such network interactions may also distinguish social cognition from other key cognitive domains such as language and attention.

These results, together with the FPCN interactions, align with a recent study that explicitly examined DMN and DAN correlation and showed that networks temporally coevolve with dependence on cognitive states, time, and subnetwork distinctions, including a strong role of FPCNs (Dixon et al. 2017).

### Fractionating and Aggregating Networks Challenge a Simple Dichotomy of Task-Positive and Task-Negative Networks

In our study, analyzing a single time series from one experiment consisting of three tasks provides a unique opportunity to identify spatiotemporal networks that emerged across all three cognitive domains and directly compare their domain-specific activity and interactions. In particular, we selected tasks that are known to evoke activity from lateral parietal nodes in the DMN. Therefore, relative to canonical resting-state networks, the 11 high-order cognitive networks we identified appear both familiar, like the bilaterally-mirrored FPCNs, DAN, and CON, and unfamiliar, because of task-driven fractionation or recruitment of specialized networks. The most prominent example of network segregation is the DMN, which was resolved into four subnetworks, labeled here as the aDMN, pDMN, temporal lobe network, and DMN. Although observations of plural DMN networks in neuroimaging experiments were initially interpreted as an artifact of analysis procedures, more recent studies investigating the functional nuances of this network support the idea of specialized subnetworks within the larger DMN (Andrews-Hanna et al. 2010; Zuo et al. 2010; Buckner and DiNicola 2019; Gordon et al. 2020). Furthermore, previous research suggests that these specialized subsystems distinctly reconfigure their interactions with other intrinsic connectivity networks across cognitive states and throughout the lifespan (Leech et al. 2011; Vatansever et al. 2015a; Davey et al. 2019; Chiou et al. 2020; Gordon et al. 2020). Extending these previous observations, we found different coupling of subnetworks across our key cognitive domains. A distinctive example includes the aDMN, whose domain-specific interactions have already been described. Another network is the semantic network, whose highest-matching network template was the Yeo 17-network parcellation’s Default B mask, followed by the general semantic cognition template from Jackson and colleagues (Yeo et al. 2011; Jackson 2021). Choosing a typical lexical decision task, we expected that ICA would specifically resolve the characteristic semantic network, so, supported by temporal behaviors characterized in our analyses, such as domain-specific deactivation and interaction with control networks, and the lack of an explicit language or semantic network in the Yeo parcellation, we labeled the component accordingly. In fact, a recent study employing procedures optimized to accomplish detailed spatial mapping in individuals characterized the language network and showed it was very closely situated to the Yeo 17-network Default B in multiple distributed regions across the cortex (Braga et al. 2020). It is well known that fMRI data analyzed at the group level loses some detail in respect to network boundaries and regions with heterogenous functions, but it is also accepted that brain function organizes in a universal structure (Biswal et al. 2010). Thus, a strength of our study is the characterization of large-scale networks and their task-specific interactions for three cognitive domains that are important in daily life. In this context, one should bear in mind that the semantic network encompasses regions of both multiple demand and default mode networks, which may reorganize differently for function in other domains (Mineroff et al. 2018; Gordon et al. 2020; Wang et al. 2021).

Notably, some of the networks we identified synchronized into cohesive units, and such spatial connectivity profiles would not have arisen in the absence of cognitive probes. For example, the ventral attention, multiple demand, and semantic networks are known to coalesce during tasks, and thorough temporal and spatial characterization of these networks is ongoing (Corbetta et al. 2008; Binder et al. 2009; Camilleri et al. 2018; Assem et al. 2020). The experimental design of this study allowed the data-driven ICA technique to explicitly resolve each of these networks, thereby substantially extending typical intrinsic connectivity results derived from resting-state experiments, and the temporal behavior of the networks reinforces distinct reorganization and coupling that arise for function across domains.

Examining the spatial topography of the individual networks in relation to one another, the networks appear closely situated and juxtaposed in a parallel arrangement in distributed regions across the cortex, which would be congruent with earlier observations in resting-state data (Yeo et al. 2014; Braga and Buckner 2017). This is most obvious for the DMN subnetworks in bilateral IPL. Moreover, there are also regions of overlap for all networks, such as the aDMN, pDMN, and DMN in the precuneus and posterior cingulate cortex. The task-active networks also exhibit parallel distribution throughout the cortex accompanied by some regions of explicit overlap. The DAN and VAN overlap in both hemispheres in posterior IPL whereas eMDN overlaps with the DAN most prominently in SPL and with the VAN more anteriorly in frontal opercula and insula cortices. Interestingly, regions in which all three networks overlap are not readily visible, and the three networks present interdigitated topologies in parietal and occipital cortices. Such overlap may be explained by intermixed neuronal populations that facilitate diverse attentional processes (Corbetta et al. 2008; Xu 2015). In particular, functional segregation of the two attention networks in the absence of a task may allow their flexible recruitment during active behavior (Corbetta et al. 2008). Another possibility that has been suggested specifically regarding the topology of eMDN is that the multiple demand system is comprised of a tightly self-connected core that universally serves domain-general purposes and a penumbra that overlaps with other networks, to facilitate information transfer between network pairs during tasks (Assem et al. 2020). In general, the overlap maps demonstrate that multiple networks simultaneously activate and deactivate within single brain regions. However, they also indicate distributed parallel mapping of cognitive function across the cortex.

In conclusion, our data provide insight into large-scale network interactions across different human-defining cognitive domains. In particular, our data show increased complexity of network interaction patterns with increasing cognitive complexity across domains. Moreover, we identified common overlapping networks that contribute to all tasks, as well as specialized configurations of network interactions, which may functionally define a particular cognitive operation. Together, these results support the flexible allocation and reallocation of different neural resources across cognitive domains distinctively facilitated through a core task-evoked architecture.

## Supplementary Material

Supplementary_Information_bhab531

## Data Availability

fMRI results and behavioral data have been previously released (Numssen et al. 2021) and are publicly available at the Open Science Framework https://doi.org/10.17605/OSF.IO/9NDHP. Subject-specific sICA and cPPI results are also available: https://doi.org/10.17605/OSF.IO/KSXQ4.
