# Supplementary material for: Task-specific network interactions across key cognitive domains
Source: Cereb Cortex. 2022 Apr 7;32(22):5050–71. doi: 10.1093/cercor/bhab531 (PMC9667178; doi:10.1093/cercor/bhab531)
Supplement: Supplementary_Information_bhab531 [file supplementary_information_bhab531.pdf]

Supplementary Information

Tables

Table S1: Spatial description and Icasso stability indices of sICA-derived networks

Component Number	Network Label	Cluster size (# voxels)	Center of Mass MNI coordinates (x,y,z)			Center Of Mass location (Eickhoff-Zilles Macro Labels)	Full network description (Eickhoff-Zilles Macro Labels)	Icasso Stability Index (I_q)	Jaccard similarity Index and template
14	Posterior default mode subnetwork (pDMN)	1223<br>4911<br>3909	-31.2<br>-34.2<br>-60.9	-30.5<br>55.8<br>38.7	32.8<br>43.1<br>-9.8	R middle frontal gyrus<br>R AG<br>R middle temporal gyrus	L/R precuneus<br>L/R middle cingulate cortex<br>L/R cuneus<br>L/R posterior cingulate cortex<br>L/R SPL<br>L superior occipital lobule	0.973	0.263, Yeo Control C (0.248, Smith DMN)
18	Temporal lobe network	870<br>638	-57<br>58	-28<br>-17	-2<br>-7	L middle temporal gyrus<br>R superior temporal gyrus	L/R middle temporal gyrus<br>L/R inferior temporal gyrus<br>L/R superior temporal gyrus<br>R medial temporal pole<br>R temporal pole	0.976	0.324, Yeo Temporo-parietal
19	Right fronto-parietal control network (rFPCN)	2426<br>1163<br>331<br>177<br>82<br>60	31<br>36<br>63<br>-42<br>-42<br>-49	31<br>36<br>63<br>-42<br>-42<br>-49	33<br>41<br>-12<br>50<br>2<br>40	R middle frontal gyrus<br>R AG<br>R middle temporal gyrus<br>L AG<br>L middle frontal gyrus<br>L middle frontal gyrus	L/R middle frontal gyrus<br>R superior frontal gyrus<br>R IFG (pars triangularis)<br>L/R superior medial gyrus<br>R IFG (pars opercularis)<br>L/R IFG (pars orbitalis)<br>L/R middle orbital gyrus<br>R anterior cingulate cortex<br>L/R AG<br>L/R IPL<br>R precuneus<br>L/R SPL<br>R supramarginal gyrus<br>R middle cingulate cortex<br>R middle temporal gyrus<br>R middle temporal gyrus<br>R inferior temporal gyrus	0.982	0.378, Smith right fronto-parietal (0.261, Yeo Control B)
28	Default mode network (DMN)	1142<br>218<br>188<br>162<br>136<br>41<br>37<br>22	-1<br>47<br>26<br>-41<br>-27<br>56<br>31<br>-8	-59<br>-70<br>15<br>-80<br>17<br>-5<br>-36<br>51	24<br>26<br>58<br>29<br>53<br>-23<br>-18<br>-9	L precuneus<br>R middle occipital gyrus<br>R superior frontal gyrus<br>L middle occipital gyrus<br>L middle frontal gyrus<br>R middle temporal gyrus<br>R fusiform gyrus<br>L mid orbital gyrus	L/R precuneus<br>L/R calcarine sulcus<br>L cuneus<br>L/R fusiform gyrus<br>L/R lingual gyrus<br>L superior occipital gyrus<br>L/R AG<br>R middle temporal gyrus<br>R middle occipital gyrus<br>L/R superior frontal gyrus<br>L/R middle frontal gyrus<br>L middle occipital gyrus<br>L IPL<br>L precentral gyrus<br>R inferior temporal gyrus<br>L/R rectal gyrus<br>L superior orbital gyrus	0.969	0.249, Smith DMN (0.191, Yeo Default C)

32	Semantic network	1145<br>968<br>237<br>166<br>128<br>63<br>32<br>21	-5<br>-46<br>46<br>-54<br>-55<br>47<br>48<br>51	30<br>18<br>28<br>-58<br>-39<br>20<br>-31<br>10	48<br>20<br>-4<br>29<br>-2<br>35<br>-4<br>-37	L superior medial gyrus<br>L IFG (pars triangularis)<br>R IFG (pars orbitalis)<br>L AG<br>L middle temporal gyrus<br>R IFG (pars opercularis)<br>R inferior temporal gyrus<br>R middle temporal gyrus	L/R superior medial gyrus<br>L/R SMA<br>L superior frontal gyrus<br>L middle frontal gyrus<br>L/R anterior cingulate cortex<br>L/R IFG (pars triangularis)<br>L/R IFG (pars orbitalis)<br>L/R middle frontal gyrus<br>L precentral gyrus<br>L/R IFG (pars opercularis)<br>L/R temporal pole<br>L AG<br>L supramarginal gyrus<br>L IPL<br>L/R middle temporal gyrus<br>L/R superior temporal gyrus<br>R inferior temporal gyrus<br>R medial temporal pole	0.953	0.267, Yeo Default B (0.220, Jackson Semantic)
35	Ventral attention network (VAN)	2249<br>2240<br>285<br>267<br>237<br>106<br>54<br>42	49<br>-20<br>-37<br>-49<br>-1<br>-33<br>-18<br>24	-9<br>-29<br>40<br>-72<br>-79<br>39<br>-5<br>-99	18<br>43<br>30<br>10<br>31<br>-16<br>74<br>-11	R rolandic operculum<br>L middle cingulate cortex<br>L middle frontal gyrus<br>L middle temporal gyrus<br>L cuneus<br>L IFG (pars orbitalis)<br>L superior frontal gyrus<br>L inferior occipital gyrus	L/R middle frontal gyrus<br>L/R supramarginal gyrus<br>L/R middle temporal gyrus<br>L/R superior temporal gyrus<br>L/R insula lobe<br>L/R IFG (pars orbitalis)<br>L/R IFG (pars triangularis)<br>R IFG (pars opercularis)<br>L/R postcentral gyrus<br>L/R temporal pole<br>R rolandic operculum<br>L/R middle occipital gyrus<br>L/R IPL<br>L/R precuneus<br>L/R middle cingulate cortex<br>L/R SMA<br>L/R SPL<br>L/R superior frontal gyrus<br>L/R middle occipital gyrus<br>L/R AG<br>L/R cuneus<br>L/R superior occipital gyrus<br>L middle orbital gyrus<br>L/R lingual gyrus<br>L/R inferior occipital gyrus<br>L/R calcarine gyrus<br>L/R inferior temporal gyrus<br>L/R cerebellum<br>L/R fusiform gyrus	0.961	0.204, Yeo Salience/ Ventral Attention A
37	Anterior default mode network (aDMN)	1730<br>358<br>206<br>205<br>164<br>117<br>98<br>82<br>66<br>27	-2<br>-2<br>-59<br>-46<br>-48<br>61<br>43<br>49<br>23<br>-25	45<br>-56<br>-9<br>34<br>-68<br>-3<br>38<br>-63<br>-42<br>-45	32<br>30<br>-24<br>-5<br>30<br>-27<br>-11<br>31<br>72<br>71	L superior medial gyrus<br>L precuneus<br>L middle frontal gyrus<br>L IFG<br>L AG<br>R middle temporal gyrus<br>R IFG (pars orbitalis)<br>R AG<br>R postcentral gyrus<br>L SPL	L/R superior medial gyrus<br>L/R superior frontal gyrus<br>L rectal gyrus<br>L/R mid orbital gyrus<br>L middle frontal gyrus<br>L/R precuneus<br>L/R middle cingulate cortex<br>L/R posterior cingulate cortex<br>L/R middle temporal gyrus<br>L/R inferior temporal gyrus<br>L medial temporal pole<br>L/R IFG (pars orbitalis)<br>L IFG (pars triangularis)<br>L temporal pole<br>L/R AG<br>R IPL<br>L/R postcentral gyrus<br>R precentral gyrus<br>L/R SPL	0.935	0.259, Yeo Default B (0.232, Yeo Default A)
41	Left fronto-parietal control network (lFPCN)	1196<br>868<br>217<br>112<br>84<br>83<br>54	-35<br>-35<br>-61<br>33<br>-4<br>-3<br>43	26<br>-67<br>-50<br>17<br>28<br>-38<br>-66	40<br>43<br>-10<br>59<br>51<br>44<br>49	L middle frontal gyrus<br>L AG<br>L middle temporal gyrus<br>R middle frontal gyrus<br>L superior medial gyrus<br>L middle cingulate cortex<br>R AG	L/R middle frontal gyrus<br>L IFG (pars triangularis)<br>L/R superior frontal gyrus<br>L middle orbital gyrus<br>L/R IPL<br>L/R AG<br>L precuneus	0.930	0.260, Smith left fronto-parietal (0.176, Yeo

							L/R SPL<br>L inferior temporal gyrus<br>L middle temporal gyrus<br>L superior medial gyrus<br>L superior frontal gyrus<br>L/R middle cingulate cortex<br>L paracentral lobule		Control B)
44	Dorsal attention network (DAN)	1948<br>1493	33<br>-31	-70<br>-78	13<br>11	R middle occipital gyrus<br>L middle occipital gyrus	L/R middle occipital gyrus<br>L/R SPL<br>L/R middle temporal gyrus<br>L/R fusiform gyrus<br>L/R lingual gyrus<br>L/R inferior occipital gyrus<br>L/R superior occipital gyrus<br>R inferior temporal gyrus<br>L IPL	0.814	0.313, Smith DAN (0.261, Yeo Dorsal Attention B)
47	(extended) Multiple demand network (eMDN)	7328<br>573<br>234<br>101<br>88<br>68<br>45<br>44<br>34<br>32<br>31<br>30	1<br>-42<br>43<br>57<br>32<br>43<br>-58<br>-16<br>-39<br>-38<br>-56<br>36	-16<br>-62<br>-71<br>-2<br>-37<br>-23<br>-38<br>66<br>-2<br>-7<br>-40<br>-2	34<br>-17<br>-14<br>-14<br>-24<br>21<br>3<br>16<br>3<br>-37<br>20<br>-43	L middle cingulate cortex<br>L fusiform gyrus<br>R inferior occipital gyrus<br>R superior temporal gyrus<br>R fusiform gyrus<br>R rolandic operculum<br>L middle temporal gyrus<br>L superior frontal gyrus<br>L insula lobe<br>L inferior temporal gyrus<br>L superior temporal gyrus<br>R inferior temporal gyrus	L/R IFG (pars triangularis)<br>L/R SPL<br>R postcentral gyrus<br>L/R precentral gyrus<br>L IPL<br>L/R middle frontal gyrus<br>L/R SMA<br>L/R IFG (pars opercularis)<br>L/R fusiform gyrus<br>L/R inferior temporal gyrus<br>L/R inferior occipital gyrus<br>L/R middle temporal gyrus<br>L/R cerebellum<br>L/R middle temporal gyrus<br>R calcarine gyrus<br>R lingual gyrus<br>L/R middle temporal gyrus<br>L/R superior temporal gyrus<br>L/R temporal pole<br>R medial temporal pole<br>L/R rolandic operculum<br>L/R supramarginal gyrus<br>L/R insula lobe<br>R Heschl's gyrus<br>L superior frontal gyrus<br>L superior orbital gyrus<br>L mid orbital gyrus<br>L superior medial gyrus	0.881	0.195, Fedorenko Multiple demand (0.180, Smith left fronto-parietal; 0.155 Yeo Control A)
49	Cingulo-opercular Network (CON)	945<br>259<br>148<br>126<br>33<br>24	0<br>-28<br>29<br>1<br>-38<br>35	42<br>46<br>47<br>-29<br>22<br>22	15<br>33<br>35<br>40<br>-8<br>-11	L anterior cingulate cortex<br>L middle frontal gyrus<br>R middle frontal gyrus<br>L middle cingulate cortex<br>L IFG (pars orbitalis)<br>R IFG (pars orbitalis)	L/R superior medial gyrus<br>L/R anterior cingulate cortex<br>L/R mid orbital gyrus<br>L/R rectal gyrus<br>L/R middle cingulate cortex<br>L/R middle frontal gyrus<br>L/R superior frontal gyrus<br>L middle orbital gyrus<br>L superior orbital gyrus<br>R paracentral lobule<br>L posterior cingulate cortex<br>L/R IFG (pars orbitalis)<br>L/R temporal pole<br>L/R insula lobe	0.681	0.190, Yeo Default A (0.154, Yeo Salience/ Ventral Attention B; 0.142, Smith cingulo-opercular)

AG, angular gyrus; IFG, inferior frontal gyrus; IPL, inferior parietal lobule; SPL, superior parietal lobule

Table S2: Statistics for domain-specific network activity

Network	Attention				Semantic				Social Cognition			
	invalid vs. rest		invalid vs. valid		word vs. rest		word vs. pseudoword		false belief vs. rest		false belief vs. true belief	
	p-value	T-value	p-value	T-value	p-value	T-value	p-value	T-value	p-value	T-value	p-value	T-value
pDMN	**3.61E-05**	-5.216	1.95E-01	-1.339	**2.35E-07**	-7.487	**5.08E-04**	4.103	7.78E-01	-0.285	2.24E-01	-1.253
Temporal	2.09E-02	-2.497	6.06E-02	-1.983	**3.25E-03**	-3.320	4.33E-01	0.800	2.63E-02	-2.390	9.45E-01	-0.070
rFPCN	**2.79E-04**	-4.353	2.18E-02	-2.478	**2.73E-06**	-6.344	8.19E-01	-0.232	**1.22E-04**	4.700	2.08E-01	-1.298
DMN	1.57E-01	-1.469	6.27E-01	0.494	**1.55E-08**	-8.858	6.45E-03	3.025	**4.12E-07**	7.218	1.80E-01	-1.388
Semantic	5.36E-02	-2.045	**2.44E-04**	-4.410	9.08E-01	-0.118	**3.14E-05**	-5.275	**1.73E-08**	8.799	5.81E-02	-2.004
VAN	2.90E-01	1.086	**8.66E-05**	-4.844	5.46E-01	0.614	3.35E-01	0.986	1.41E-02	-2.676	6.00E-01	0.532
aDMN	7.20E-01	-0.363	6.89E-02	1.917	**1.50E-08**	-8.874	**1.38E-04**	4.649	**3.07E-11**	-12.573	3.62E-01	0.931
lFPCN	1.47E-02	-2.659	1.08E-01	-1.678	**1.02E-08**	-9.080	1.15E-01	1.644	**2.87E-05**	5.314	9.84E-02	-1.729
DAN	3.39E-02	2.269	**2.40E-03**	-3.449	3.35E-01	0.987	1.36E-01	-1.552	**3.45E-11**	12.494	1.54E-01	1.478
eMDN	**5.99E-08**	-8.160	9.77E-01	0.029	**2.72E-06**	6.347	**2.39E-06**	-6.403	3.84E-02	-2.209	6.32E-01	-0.486
CON	**3.80E-04**	-4.224	6.38E-01	0.478	3.61E-02	-2.239	5.29E-02	2.051	**1.07E-11**	-13.300	9.47E-01	-0.067

Bold values indicate significant comparisons in the domain-specific two-sided t-tests (Bonferroni-corrected $p < 0.05$); pDMN, posterior default mode network; rFPCN, right fronto-parietal control network; DMN, default mode network; VAN, ventral attention network; aDMN, anterior default mode network; lFPCN, left fronto-parietal control network; DAN, dorsal attention network; eMDN, extended multiple demand network; CON, cingulo-opercular network.

Figures

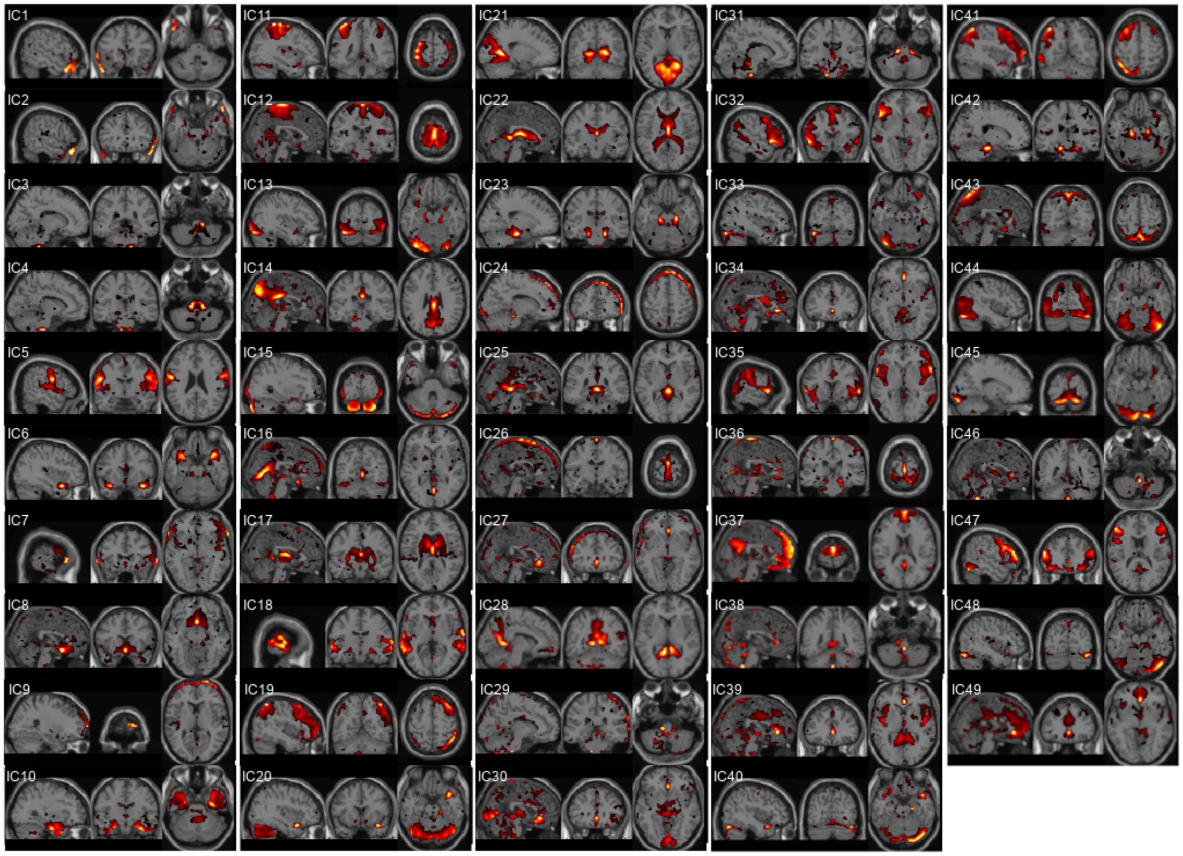

Figure S1: 49 components resolved with sICA

Full set of components resolved from the data with sICA include noise and neural activity components. Orthogonal brain slices are displayed at each component's maximum intensity location. Z-scores are displayed and scaled for each component.

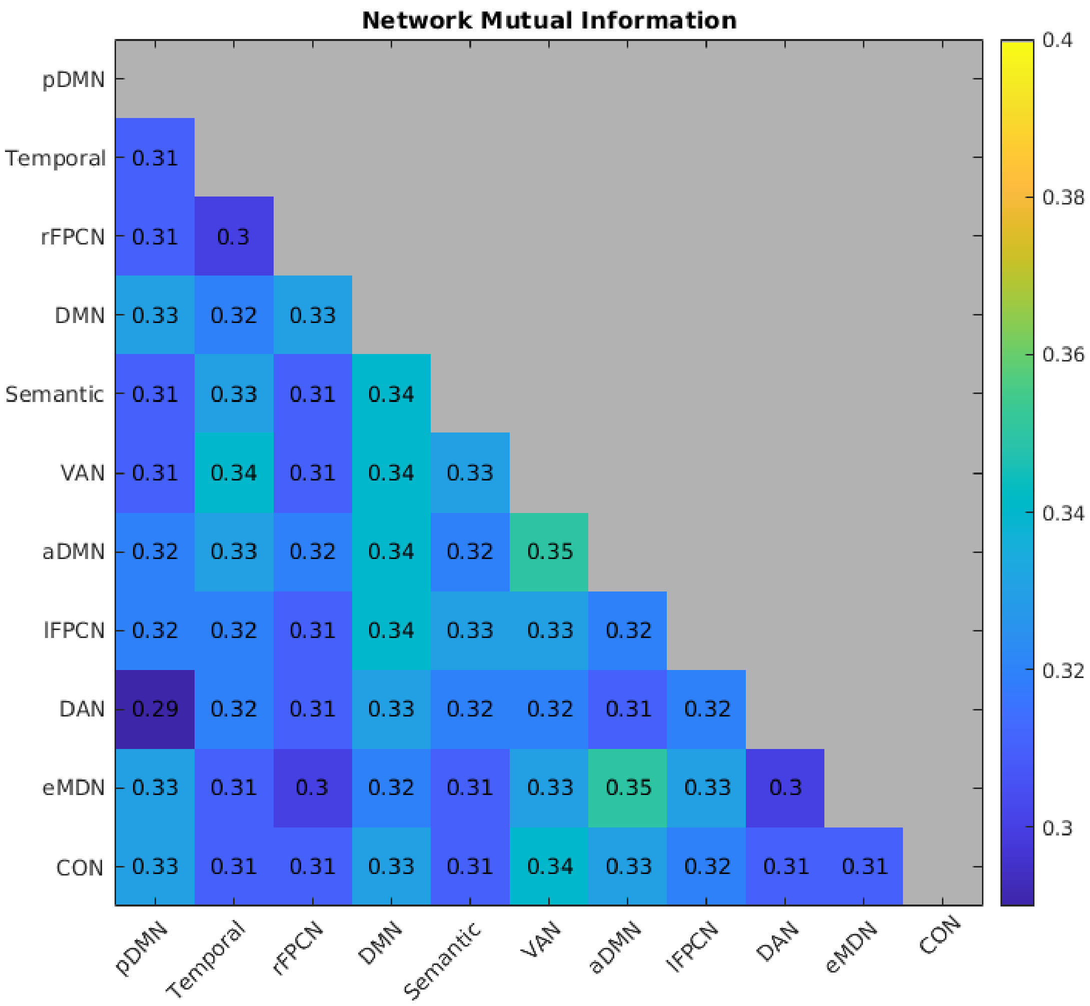

Figure S2: Selected network spatial correlations

Low correlations between networks show that they are spatially distinct. Spatial correlations (r) are displayed for the examined networks. Network maps were thresholded by one-sided t-test results at FDR-corrected p ≤ 0.05 then correlated within a cerebrum mask.

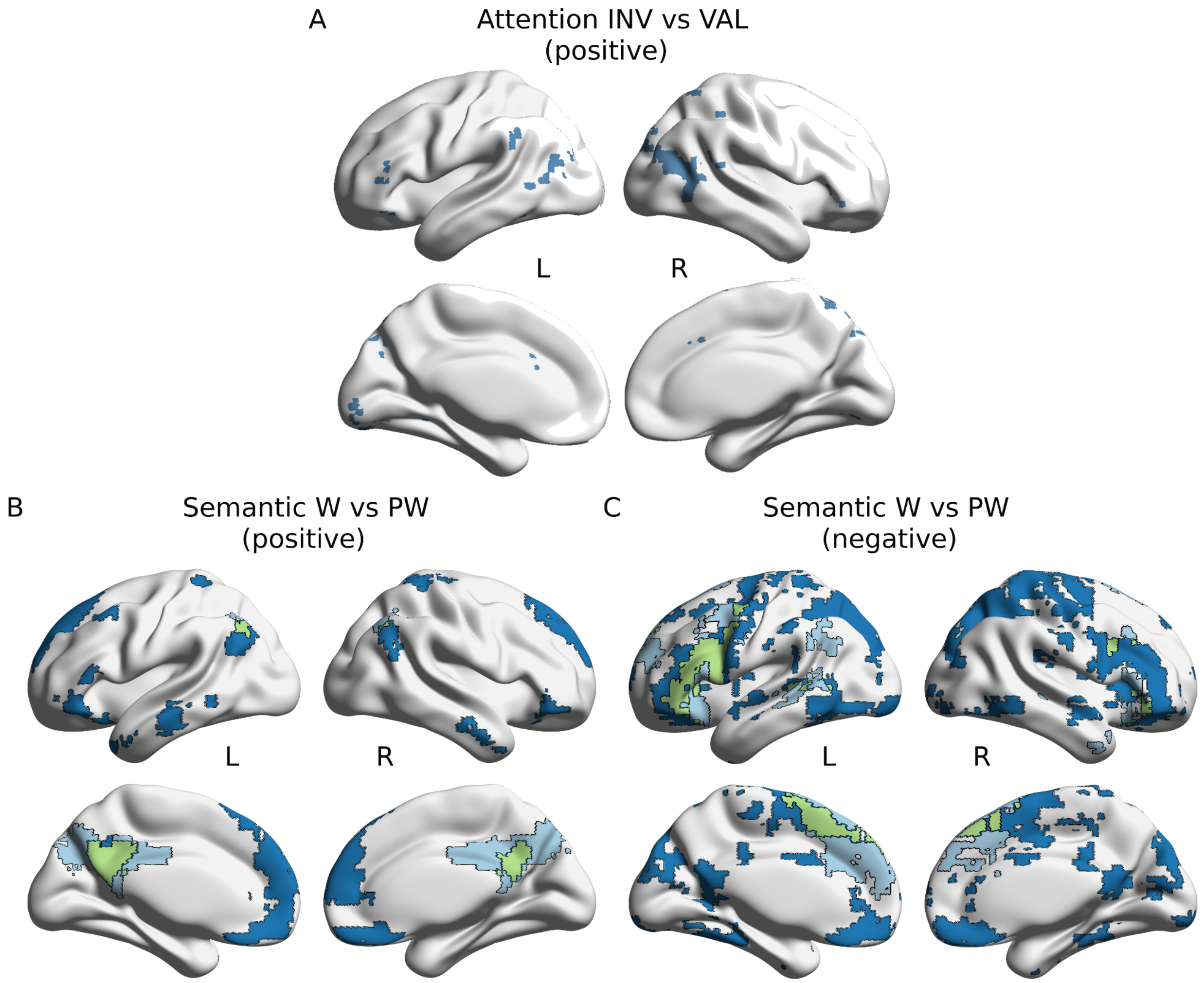

Figure S3: Domain-specific network overlap for target-control comparisons

Overlap maps of networks that were significantly active in the target-control comparisons for each domain. (A) Attention, portrayed as in main text Figure 4, shows regions in which two positively active networks in the invalid vs. valid comparison overlap; the map comprises three active networks. The lower panels in the figure adopt a different depiction scheme in which two positively (B) or negatively (C) active networks comprise the overlap in the word versus pseudoword comparison for semantics, so each network is explicitly displayed as blue or light blue, with green representing the overlap of the two networks.

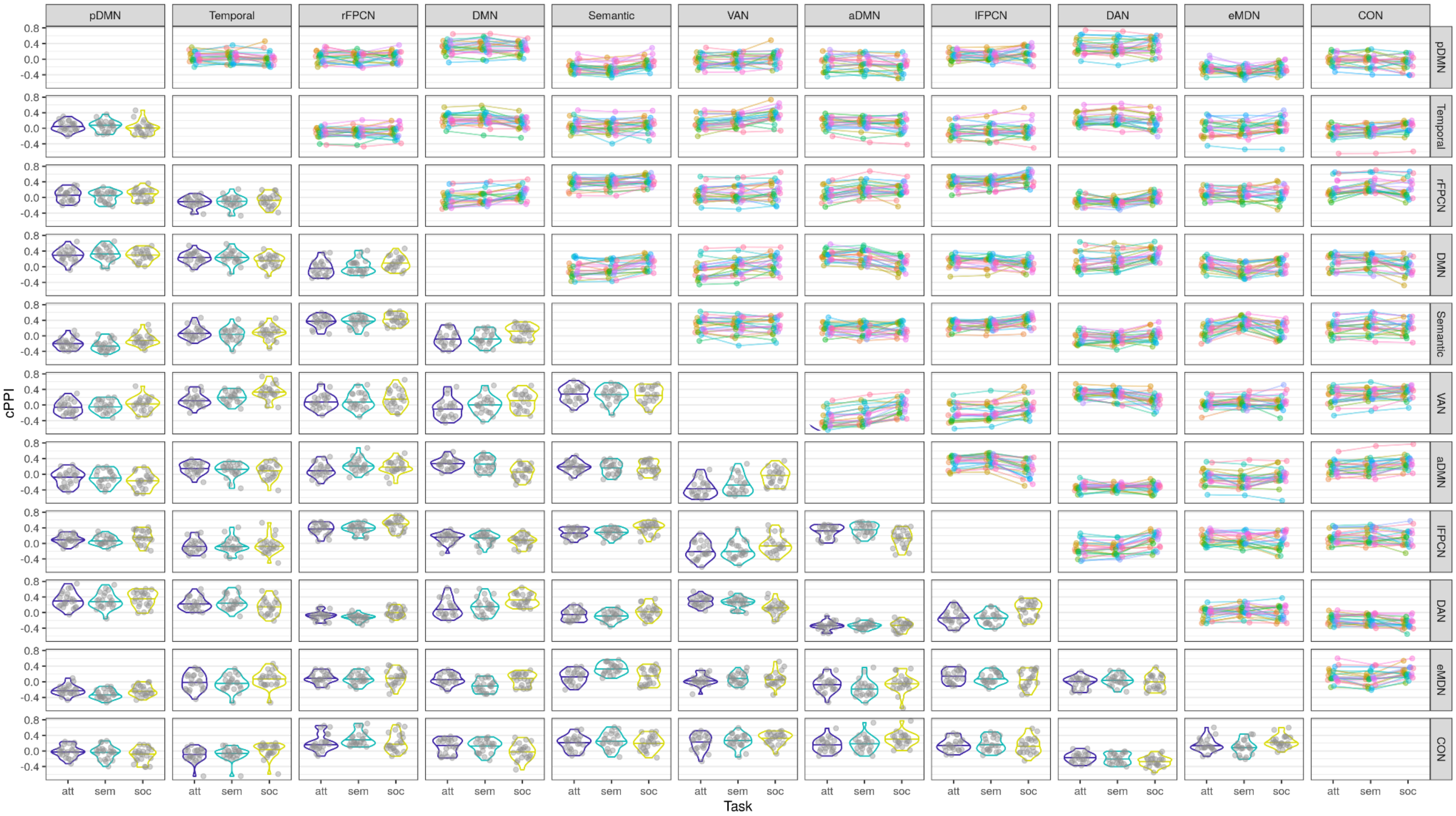

Figure S4: Subject-specific network interaction (cPPI) by network and task

Violin plots(lower left triangle) show variability of network interactions across subjects. Line plots (upper right triangle) emphasize network coupling patterns that behave with domain specificity and are generally consistent across subjects.
